# Two Ecdysteroids Isolated from Micropropagated *Lychnis flos-cuculi* and the Biological Activity of Plant Material

**DOI:** 10.3390/molecules26040904

**Published:** 2021-02-09

**Authors:** Michał P. Maliński, Jaromir Budzianowski, Małgorzata Kikowska, Monika Derda, Marcelina M. Jaworska, Dariusz T. Mlynarczyk, Marta Szukalska, Ewa Florek, Barbara Thiem

**Affiliations:** 1 Chair and Department of Pharmaceutical Botany and Plant Biotechnology, Poznan University of Medical Sciences, 14 Sw. Marii Magdaleny St., 61-861 Poznań, Poland; jbudzian@ump.edu.pl (J.B.); kikowska@ump.edu.pl (M.K.); bthiem@ump.edu.pl (B.T.); 2 Chair and Department of Biology and Medical Parasitology, Poznan University of Medical Sciences, 10 Fredry St., 61-701 Poznań, Poland; mderda@ump.edu.pl; 3 Chair and Department of Genetics and Pharmaceutical Microbiology, Poznan University of Medical Sciences, 4 Święcickiego St., 60-781 Poznań, Poland; marcelinajaworska@ump.edu.pl; 4 Chair and Department of Chemical Technology of Drugs, Poznan University of Medical Sciences, 6 Grunwaldzka St., 60-780 Poznań, Poland; mlynarczykd@ump.edu.pl; 5 Laboratory of Environmental Research, Chair and Department of Toxicology, Poznan University of Medical Sciences, 30 Dojazd St., 60-631 Poznań, Poland; martan@ump.edu.pl (M.S.); eflorek@ump.edu.pl (E.F.)

**Keywords:** 20-hydroxyecdysone, polypodine B, Ragged Robin, plant tissue culture, *Acanthamoeba*, antifungal activity, Microtox

## Abstract

Genetically uniform plant material, derived from *Lychnis flos-cuculi* propagated in vitro, was used for the isolation of 20-hydroxyecdysone and polypodine B and subjected to an evaluation of the antifungal and antiamoebic activity. The activity of 80% aqueous methanolic extracts, their fractions, and isolated ecdysteroids were studied against pathogenic *Acanthamoeba castellani*. Additionally, a Microtox^®^ acute toxicity assay was performed. It was found that an 80% methanolic fraction of root extract exerts the most potent amoebicidal activity at IC_50_ of 0.06 mg/mL at the 3rd day of treatment. Both ecdysteroids show comparable activity at IC_50_ of 0.07 mg/mL. The acute toxicity of 80% fractions at similar concentrations is significantly higher than that of 40% fractions. Crude extracts exhibited moderate antifungal activity, with a minimum inhibitory concentration (MIC) within the range of 1.25–2.5 mg/mL. To the best of our knowledge, the present report is the first to show the biological activity of *L. flos-cuculi* in terms of the antifungal and antiamoebic activities and acute toxicity. It is also the first isolation of the main ecdysteroids from *L. flos-cuculi* micropropagated, ecdysteroid-rich plant material.

## 1. Introduction

*Lychnis flos-cuculi* L. [*Silene flos-cuculi* (L.) Greuter & Burdet, *Coronaria flos-cuculi* (L.) A. Braun], commonly known as Ragged Robin, is a perennial herb native to Europe and Asia, spreading through North America. While still a common species in meadows and wetlands, the amelioration of its habitat for agricultural purposes has shrunk its populations. The medicinal applications of the species by folk medicine include wound healing, the treatment of headaches, stomach pain, and interestingly, malaria, but there are no modern medicinal applications for Ragged Robin. Until recently, little was known about the phytochemical constituents of this plant. However, it is now acknowledged that it contains phytoecdysteroids, triterpenoid saponins, flavonoids, and phenolic acids [1,2,3,4,5,6]. The species was introduced to in vitro cultures by our research team and the protocols of micropropagation and callus induction and development were established for the first time. Various in vitro systems, accumulating enhanced amounts of ecdysteroids, especially mature micropropagated plants, were established by Maliński et al. [6].

Among the secondary metabolites of Ragged Robin, ecdysteroids stand out as compounds with unique properties. Their role as phytoalexins is to deter insects from feeding on plants, as phytoecdysteroids are structurally identical or similar to arthropod molting hormones (zooecdysteroids), having severe detrimental effects on insect physiology. Several taxa are particularly rich in these polyhydroxylated steroids, as they were found in angiosperm families Caryophyllaceae, Amaranthaceae, Chenopodiaceae, Asteraceae, and Lamiaceae. Especially worth mentioning is the genus *Silene*, interrelated to *Lychnis,* in which diverse ecdysteroids are present in high quantities. In these plants, ecdysteroids can reach concentrations of ca. 1–2% of the plant’s dry weight. However, the ecdysteroid content in plants is often too low for practical use, unpredictably variable, and highly dependent on numerous environmental factors. Therefore, the introduction of ecdysteroid-producing species into controlled in vitro conditions allows uniform plant material, often richer in secondary metabolites, to be obtained [7]. In the plant kingdom, the most predominant ecdysteroid is 20-hydroxyecdysone (20E), while polypodine B (polB) is among the most common. Both are dominant ecdysteroids in *L. flos-cuculi* [6]. The adaptogenic, anabolic, and neuroprotective properties of ecdysteroids have been widely reported and reviewed [8,9,10]. Their multidirectional activity also includes an antimicrobial effect, which, according to some studies, is especially attributed to less polar derivatives [11]. The mild antioxidant properties of ecdysteroids have also been reported, but manifesting through cell signaling or enzyme inhibition, leading to an antioxidant response in live cells, rather than radical scavenging [12]. In fact, the postulated influence on intracellular signaling, including protein kinases and transcription factors, is the likely reason behind their hypoglycemic, hypocholesterolemic, neuroprotective, and anti-apoptotic effect. Their anabolic activity is a result of a direct increase of mRNA translation, unrelated to androgen receptor agonism. Ecdysteroids promote the healing of wounds and burns. Additionally, their toxicity in mammals is exceptionally low. On the other hand, they seem to be toxic to microorganisms, including bacteria, fungi, and some protozoa [8,9].

There is an ever-present interest in searching for new antibacterial and antifungal agents, as pathogenic microorganisms may quickly gain resistance against currently used chemotherapeutics. Fungal infections of skin and mucous membranes or pulmonary mycoses are a common problem, especially when natural human microflora is disturbed. The structural diversity of biocidal substances in natural products, and often their synergistic effect, are an advantage, hindering the development of drug resistance by microorganisms [13].

*Acanthamoeba* is a genus of opportunistic pathogenic amoebae. It is widely found in the natural environment, inhabiting soil, water, and air. This unicellular eukaryote exists as either dormant cysts or vegetative trophozoites, which are the infective forms. Their portal of entry is through the damaged epithelium or eyes, causing amoebic keratitis affecting the skin and cornea, which may lead to severe complications, including potentially fatal granulomatous amoebic encephalitis. The treatment is difficult and not always effective due to resistance, while the antibiotics used exert many adverse effects and are inherently toxic. Therefore, many substances of plant origin are being investigated for antiamoebic activity, in order to apply them as combined therapy and reduce the effective dose of antibiotics [14,15,16].

The antimicrobial effect of many natural compounds, especially sterols or saponins able to disrupt the integrity of cellular membranes, often extends to biocidal activity against unicellular parasites, such as pathogenic amoebae [17]. This prompted us to evaluate the potential amoebicidal effect of secondary metabolites present in Ragged Robin, including isolated ecdysteroids.

This study is a continuation of a larger project on *Lychnis flos-cuculi*. This taxon was introduced to in vitro conditions by our team. Then, various in vitro systems were established and stabilized for the first time. Previously, it was reported that the flowering herb and roots of micropropagated plants transferred into soil are especially high in 20-hydroxyecdysone and polypodine B. The stabilized callus was rich in triterpenoid saponins, though unable to produce ecdysteroids. The total phenolic, total phenolic acid, and total flavonoid content in the resulting diverse types of biomass were evaluated and the antioxidant capacity was compared by 2,2-diphenyl-1-picrylhydrazyl radical (DPPH) and ferric reducing antioxidant potential (FRAP) assays. The flowering herb and roots of the micropropagated plants transferred to the soil, as well as the selected callus, were characterized by phytochemical screening of 80% methanolic extracts of the plant. These results enhanced the knowledge on Ragged Robin’s chemical profile [6,18].

The current study focuses further on the aforementioned materials of in vitro origin. The aim of this work was to isolate two main ecdysteroids—20-hydroxyecdysone and polypodine B—from the flowering herb of *L. flos-cuculi* propagated in vitro, and evaluate their antiamoebic activity and acute toxicity using a Microtox^®^ assay, in comparison with those of extracts and their fractions derived from callus and flowering herbs and roots of plants after micropropagation. As an additional step, the antifungal activity of extracts against pathogenic species of fungi was evaluated using the serial microdilution method.

## 2. Results

### 2.1. Preparation of Extracts and Fractionation

This study is a continuation of a our larger project on *Lychnis flos-cuculi,* regarding introduction of the plant to in vitro cultures, the development of diverse in vitro systems, phytochemical analysis of the obtained biomass, and evaluation of the antioxidant activity. In previous studies, it was reported that the flowering herb and roots of micropropagated plants transferred into soil are especially high in 20-hydroxyecdysone and polypodine B. The content of ecdysteroids is almost two times higher than in intact plants: 20E present at 4.8 mg/g and polB at 3.9 mg/g in terms of the dry weight of the herb, whereas roots contain 20E at 3.7 mg/g and polB at 3.2 mg/g of dry weight. The callus rich in triterpenoid saponins, though unable to produce ecdysteroids, was obtained [6,18].

In this work, the methanolic percolate of flowering herb was prepared to isolate the main ecdysteroids. For the evaluation of biological activity, the flowering herb, roots, and callus were extracted with 80% aqueous methanol and the extracts were fractionated by solid phase extraction with different aqueous methanol solutions. The resulting 40% and 80% methanolic fractions and isolated compounds, along with crude unfractionated extracts, were tested for antiamoebic activity. Fractions and isolated ecdysteroids were tested for acute toxicity. For antifungal activity, a crude methanolic extract was used.

### 2.2. Isolation and Structural Elucidation of Ecdysteroids

The compounds 20-hydroxyecdysone and polypodine B were isolated from methanolic percolate from the flowering herb of micropropagated *Lychnis flos-cuculi*.

Compounds **1** and **2** were identified as 20-hydroxyecdysone (20E) and polypodine B (polB), respectively (Figure 1, Table 1), based on their NMR spectral data (^1^H NMR, ^13^C NMR, ^1^H-^1^H COSY, ^1^H-^13^C HSQC and ^1^H-^13^C HMBC, and NOESY) (Figures S1–S12, Supplementary Material) in comparison with those reported in the literature [19,20,21,22,23].

Compound **1** was isolated as a white powder and its HR-MS (MALDI) spectrum gave a pseudo-molecular ion [M + K]^+^ at *m*/*z* 519.2788 corresponding to the formula C_27_H_44_O_7_. The compound exhibited signals of 27 carbon nuclei in the ^13^C NMR spectrum, including downfield shifted signals due to the presence of a carbonyl at 206.5 ppm; an olefinic bond at 168.0 and 122.2 ppm; and substitution by six hydroxyl groups at 85.3, 78.5, 77.9, 71.3, 68.7 and 68.5 ppm. Moreover, the ^1^H NMR spectrum showed resonances of five methyl groups, each as a singlet integrated for three protons (3H) at 0.88, 0.91, 1.19, 1.18, and 1.20 ppm, as well as an olefinic proton at 5.80 ppm (d, *J* = 2.5 Hz). The olefinic proton (H-7) signal at 5.80 ppm was a doublet due to allylic coupling (*J* = 2.6 Hz) with H-9 at 3.15 ppm. The low-field proton signal at 3.94 ppm due to the substitution with an OH group exhibited ^1^H-^1^H COSY couplings to methylene protons at 1.72 and 1.69 ppm, and the latter signals showed long-range HMBC ^3^*J*_HC_ couplings to the carbonyl carbon (C-6) signal at 206.5 ppm. Therefore, the 3.94 ppm signal was assigned to H-3 and the methylene to H-4. Moreover, the H-3 signal was a narrow multiplet indicative of the equatorial orientation, which was revealed by small vicinal couplings (*J* = ca 3 Hz) to oxymethine (at 3.83 ppm; ddd, *J* = 11.9, 4.4, 3.1 Hz) and methylene (at 1.78 m and 1.42 m ppm) protons in the ^1^H-^1^H COSY spectrum assigned to C-2 and C-1 positions, respectively. The H-5 proton signal at 2.37 ppm (dd) had an axial orientation evidenced by diaxial (*J* = 12.9 Hz) and axial-equatorial (*J* = 4.5 Hz) couplings to H-4 protons. The interactions observed in the NOESY spectrum exhibited spatial proximity between axial H-1 (1.42 ppm), H-5, and C-19 methyl protons (0.96 ppm; s), as well as between H-2 and H-9, and thus indicated a *cis* junction of A and B rings.

Compound **2** was isolated as a white powder and its HR-MS (MALDI) spectrum gave a pseudo-molecular ion [M + Na]^+^ at *m*/*z* 519.2921 corresponding to the formula C_27_H_44_O_8_. The compound showed NMR spectra very similar to those of compound **1**, with the exception of the presence of one additional hydroxyl group attached to the carbon with a downfield shift signal at 80.3 ppm and the absence of the H-5 signal observed in the ^1^H NMR spectrum of compound **1** at 2.37 ppm. The H-3 signal was recognized in the ^1^H NMR spectrum as a narrow multiplet at 3.98 ppm (ddd, *J* = 3.4, 3.1, 3.1 Hz) from its HMBC coupling to C-5 (80.3 ppm) and ^1^H-^1^H COSY coupling to H-2 oxymethine at 3.94 ppm (ddd, *J* = 10.3, 6.5, 3.1 Hz). The NOESY interactions were observed between an axial H-1 (1.73 ppm; m) and C-19 methyl (0.91 ppm; s), as well as H-2, an axial H-4 (2.07 ppm; dd, *J* = 14.7, 3.1 Hz), and H-9 (3.18 ppm; ddd, *J* = 10.8, 7.2, 2.6). Those data pointed to the *cis* junction of A and B rings, which requires a β configuration of the OH group at C-5 in compound **2**. All 1D and 2D NMR spectra only showed signals assignable to the investigated compounds **1** and **2**, thus pointing to their high purity, being significantly higher than 95%. HPLC analyses of the purified compounds were performed using a diode array detector (DAD), indicating a 98.8% purity for compound **1** and 99.1% for compound **2** (Supplementary Material, Figures S18–S19).

### 2.3. Antifungal Activity Screening

Methanolic extracts from the callus, flowering herb, and roots of *L. flos-cuculi* were used for the preliminary screening of antifungal activity. The tested extracts indicated similar, moderate antifungal activity, with minimum inhibitory concentration (MIC) values ranging from 1.25 to 2.5 mg/mL. *Cryptococcus neoformans* was the species most sensitive to the studied extracts. The lowest MIC values were observed for the extract prepared from roots against *Cryptococcus neoformans* and *Aspergillus brasiliensis* (Table 2).

### 2.4. Antiamoebic Activity of Isolated Compounds and Plant Material Fractions

For this assay, the samples consisted of 40% and 80% fractions derived from 80% aqueous methanolic extracts of flowering herb, root, and callus, along with unfractionated extracts from herb and root, and isolated ecdysteroids **1** and **2**.

**Figure 2:**
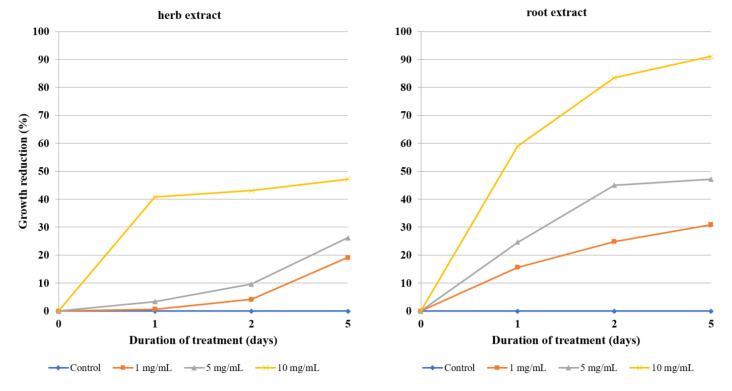
The effect of unfractionated 80% aqueous methanolic extracts from the herb and roots of *Lychnis flos-cuculi* on the inhibition of *Acanthamoeba* trophozoite proliferation in the culture medium.

All of the studied samples demonstrated time- and dose-dependent amoebicidal activity on the trophozoites. The crude, unfractionated extracts exhibited relatively weak activity (Table 3, Figure 2), requiring a high concentration (10 mg/mL) to exert a noticeable effect on trophozoite proliferation. Even at this concentration, the herb extract did not reach 50% growth reduction. The root extract was more potent, causing close to 50% growth reduction at 5 mg/mL, and the treatment with 10 mg/mL over 5 days reduced the amoeba proliferation by 90%.

**Figure 3:**
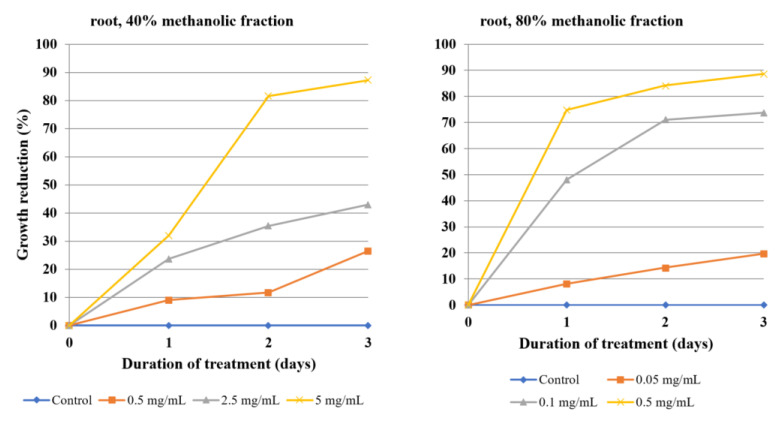
The effect of the 40% and 80% methanolic fractions of extract from the roots of *Lychnis flos-cuculi* on the inhibition of *Acanthamoeba* trophozoite proliferation in the culture medium.

**Figure 4:**
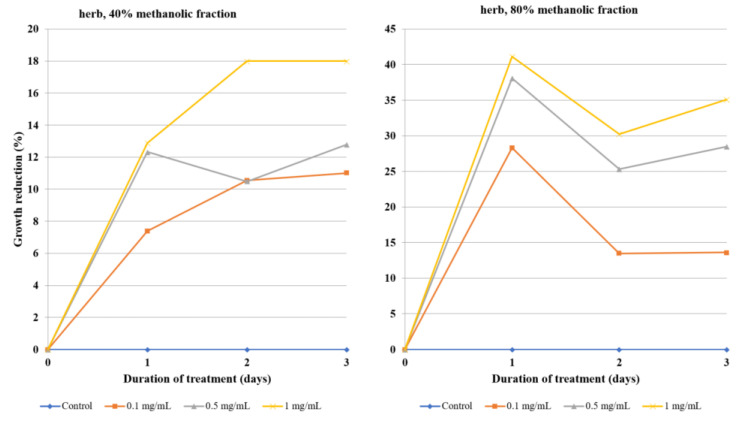
The effect of the 40% and 80% methanolic fractions of extract from the flowering herb of *Lychnis flos-cuculi* on the inhibition of *Acanthamoeba* trophozoite proliferation in the culture medium.

**Figure 5:**
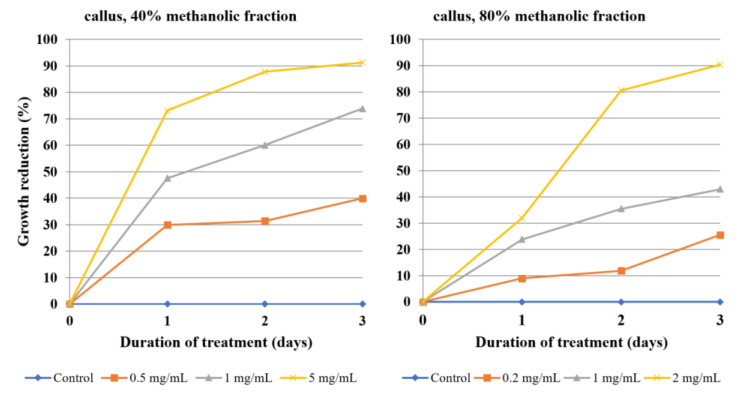
The effect of the 40% and 80% methanolic fractions of *Lychnis flos-cuculi* callus extract on the inhibition of *Acanthamoeba* trophozoite proliferation in the culture medium.

The antiamoebic activity of 80% methanolic fractions derived from the callus, herb, and roots was noticeably higher than that of the 40% fractions. The 80% fraction of root extract was especially effective, as the concentration of 0.1 mg/mL was able to reduce the growth of *Acanthamoeba* by almost half after the first day of incubation and by 75% after day three. A higher concentration, i.e., 0.5 mg/mL, reached 75% growth reduction after the first day and about 90% after day three (Table 4, Figure 3). Fractions derived from flowering herb affected amoeba the least, with the 50% growth inhibition threshold not being reached, even at the highest tested concentrations (Table 5, Figure 4). On the other hand, callus fractions at the highest concentration studied exhibited 90% growth reduction. Interestingly, at the concentration of 1 mg/mL, the 40% fraction was found to inhibit *Acanthamoeba* trophozoites more efficiently than the 80% fraction (Table 6, Figure 5).

**Figure 6:**
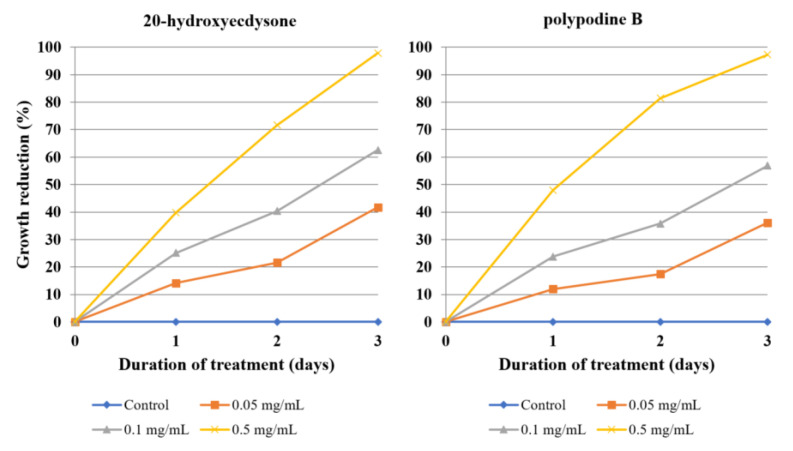
The effect of 20-hydroxyecdysone and polypodine B isolated from *Lychnis flos-cuculi* L. on the inhibition of *Acanthamoeba* trophozoite proliferation in the culture medium.

**Figure 7:**
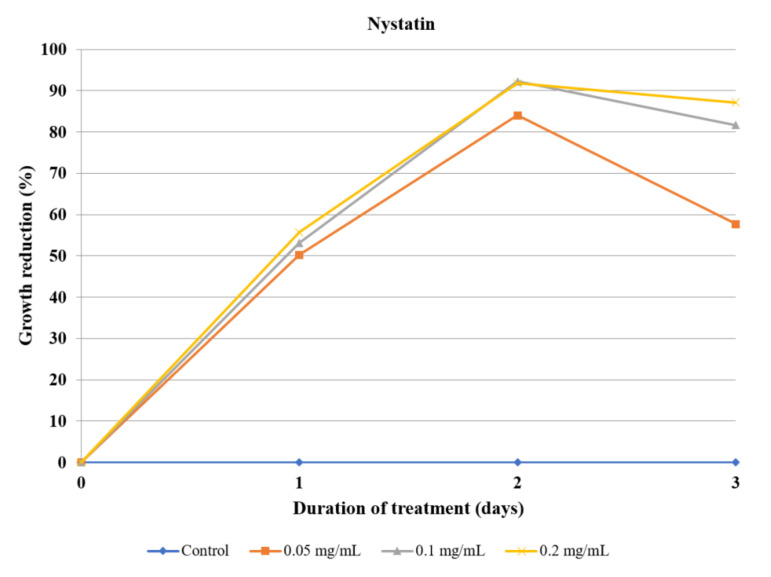
The effect of nystatin used as a positive control on the inhibition of *Acanthamoeba* trophozoite proliferation in the culture medium.

The two isolated ecdysteroids revealed almost identical effectivity on each day of treatment, with 20-hydroxyecdysone being marginally stronger. At a 0.5 mg/mL concentration, both compounds were able to almost completely inhibit the amoeba growth after three days, while reducing it by half after day one (Table 7, Figure 6). Nystatin was used as a positive control (Figure 7, Table S1, Supplementary Material).

The IC_50_ (inhibitory concentration causing 50% growth inhibition) values calculated for each day clearly show the very high potency of the 80% methanolic fraction from the root extract, with IC_50_ of 0.06 mg/mL. This parameter is similarly high for both 20-hydroxyecdysone and polypodine B (0.07 mg/mL). The IC_50_ for the 40% methanolic fraction of the callus extract (0.55 mg/mL) is lower than that for the 80% fraction (1.15 mg/mL). Values calculated for the remaining studied fractions are above 1 mg/mL, with the highest IC_50_ for the 40% methanolic fraction for the root extract at 2.95 mg/mL (Table 8).

### 2.5. Microtox Assay

**Figure 8:**
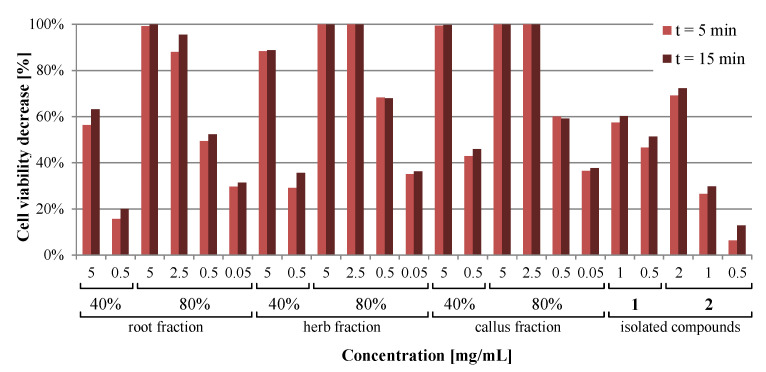
Comparison of the acute toxicity of 40% and 80% aqueous methanolic fractions from *Lychnis flos-cuculi* extracts and isolated ecdysteroids after 5 and 15 min, measured using the Microtox assay.

An acute toxicity test was performed on the same set of samples that were evaluated for antiamoebic activity, except for the crude extracts. The results of the assay indicate that 80% methanolic fractions from either callus, herb, or root were clearly the strongest agents, as they induced the highest response. Toxicity (interpreted as an *Aliivibrio fischeri* cell viability decrease of at least 20%) was observed at a concentration of 0.05 mg/mL. There are minor differences between the fractions given the source of plant material, suggesting that flowering herb is the most potent, followed by callus, and root is the weakest. The toxicity of 40% fractions is far weaker, with a 10-fold higher concentration (0.5 mg/mL) being required to exert a comparable effect. The root fraction was again the least toxic, bordering on a 20% threshold, while herb and especially callus-derived fractions were considerably stronger. The isolated ecdysteroids only displayed moderate toxicity in this model, as 20-hydroxyecdysone decreased the cell viability by approximately half at the concentration of 0.5 mg/mL, while polypodine B caused borderline toxicity at 1 mg/mL (Figure 8, Table S2, Supplementary Material).

### 2.6. Ecdysteroid Content in Tested Samples

The content of 20-hydroxyecdysone and polypodine B in crude extracts and fractions used for bioassays was measured using the HPLC-DAD method employed in our previous work to quantify the ecdysteroids in *L. flos-cuculi* material of diverse origins [6]. The results are summarized in Table 9.

Based on the quantification of ecdysteroids in extracts and fractions, the concentration of ecdysteroids in sample dilutions used for bioassays was calculated. The results are presented in Table S3 (Supplementary Material).

## 3. Discussion

The isolation protocol focuses on two main ecdysteroids of the species and therefore, is a simplified, modified version of other known protocols of ecdysteroid isolation [22,24]. Dichloromethane-water partition is suitable for the separation of chlorophyll and other lipophilic compounds. The partition coefficients of compounds **1** and **2** are both favorable enough to keep them out of the dichloromethane phase (content below the thin layer chromatography (TLC) detection threshold). Similarly, no ecdysteroids are detectable by TLC in the aqueous (II) phase. Though this method would be unsuitable for the isolation of less hydrophilic ecdysteroids, including esters and ketonides, where hexane is most often used for initial liquid–liquid partition [23], it still constitutes a relatively simple isolation protocol for the most common ecdysteroids, including the most extensively studied 20-hydroxyecdysone. The analytical purity of the ecdysteroids efficiently separated by preparative TLC was not reached with the C-18 stationary phase eluted with a water–methanol gradient. However, final purification using aluminum oxide, efficient for getting rid of compounds with aromatic 5-hydroxy-4-keto or *ortho*-dihydroxy functions in our previous studies [25], worked sufficiently for that purpose.

The identity of the compounds was confirmed using a series of physicochemical characterization techniques, including mass spectrometry (MALDI), NMR, UV-Vis, and FT-IR spectroscopy. In the case of the NMR spectra, the signals in ^1^H NMR and ^13^C NMR were assigned with the aid of 2D NMR spectra, confirming the structure of isolated compounds as 20-hydroxyecdysone and polypodine B, considering their relative stereochemistry, including the *cis* junction of rings A and B. In UV-Vis spectra, the absorption maximum for **1** in methanol was observed at 242 nm (logε = 4.07) and for **2** in ethanol at 237 nm (logε = 4.03), which is consistent with the literature data [26,27]. The analysis of the signals found in the IR spectra correlated well with the values for the functional groups found in the structure of these ecdysteroids (supplementary data). The mass spectrometry further confirmed the elemental composition of the isolated compounds, as the recorded MALDI HR mass spectra revealed pseudo-molecular peaks: [M + K]^+^ at *m*/*z* 519.2788 for **1** and [M + Na]^+^ at *m*/*z* 519.2921 for **2** confirming formula C_27_H_44_O_7_ and C_27_H_44_O_8_, respectively.

Although the absolute stereochemistry of compounds **1** and **2**, which can be crucial for the biological activity, was not determined, it is assumed that they are 2*S*,3*R*,14*S*,20*R*,22*R*,25-hexahydroxy-5*S*-cholest-7-en-6-one (2β,3β,14α,20*R*,22*R*,25-hexahydroxy-5β-cholest-7-en-6-one) and 2*S*,3*R*,5*S,*14*S*,20*R*,22*R*,25-heptahydroxy-5*S*-cholest-7-en-6-one (2β,3β,5β,14α,20*R*,22*R*,25-heptahydroxy-5β-cholest-7-en-6-one), respectively, as established by X-ray crystallographic studies [28], which confirmed an earlier established structure of an ecdysone skeleton [29]. Additionally, one general route of ecdysone biosynthesis in plants has been established without variability in the stereochemistry, except for the *trans* or *cis* junction of A/B rings [10,30]. Moreover, any epimeric form would be recognized by an apparent change in the multiplicity and magnitude of coupling constants of a methine ^1^H NMR signal at a chiral center, and a chemical ^13^C NMR shift of the vicinal carbon signal, as exemplified by 22*R* and 22*S* epimers of 20-hydroxyecdysone [31].

To the best of our knowledge, there are no other studies regarding the biological activity of *Lychnis flos-cuculi* in the scientific literature, except for reports of antibacterial activity by Mamadalieva et al. [11] and an evaluation of the antioxidant capacity by Maliński et al. [18].

The results of our recent study [18] include the phytochemical screening of the callus, herb, and root of micropropagated plants, revealing complex oleanane-type triterpenoid saponins as the dominant group of metabolites in all plant materials. Diverse glycosides of gypsogenin and gypsogenic, oleanolic, and quillaic acids were detected. In addition, the saponins present in each plant material had different structures. Ecdysteroids were the most structurally diverse in roots, including 20E and polB, as well as ecdysone, ajugasterone C, integristerone A, and viticosterone E (an acetyl derivative) as minor constituents. The only ecdysteroids detected in flowering herb were 20E and polB, while none of them were found in the callus. The occurrence of flavonoid compounds is practically limited to the flowering herb, and these include *C*-glycosyls and *O*-glycosides of apigenin and luteolin. Flavonoids with two glycoside groups, or additional acetylated sugar moieties, are present among them [18]. The preliminary TLC analysis of fractions used in this study revealed the presence of saponins in 80% fractions. Meanwhile, the 40% fractions mostly consisted of the polyphenolic compounds. The quantitative evaluation of the ecdysteroid content in studied fractions by HPLC showed that 80% fractions, especially the root fraction, are richer in ecdysteroids compared to 40% fractions (Table 9). The analysis confirmed that callus fractions do not contain 20-hydroxyecdysone or polypodine B.

The preliminary assay for the antifungal activity of methanolic extracts from the herb and root of micropropagated plants from the experimental plot and callus served as an initial screening to estimate the extent of their biological activity. The extracts from *L. flos-cuculi* demonstrated moderate and relatively uniform antifungal activity, with an MIC in range of 1.25–2.5 mg/mL. Similar antifungal activity assays performed on methanolic extracts from leaves and roots of *Eryngium planum*, *Eryngium campestre*, and *Eryngium maritimum* demonstrated less potent antifungal activity against *Candida albicans* (MIC above 12.5 mg/mL) and *Aspergillus niger* (MIC above 25 mg/mL) [32]. Interestingly, aqueous ethanolic extracts from the same material exerted significantly higher activity, with the MIC ranging from 0.04 to 1.9 mg/mL, especially against *Trichophyton mentagrophytes* [33].

Minor differences between the MIC of studied extracts suggest that the amount and diversity of ecdysteroids do not affect the antifungal activity. Roots contain multiple ecdysteroids, including acyl derivatives, the 20E and polB were the only ecdysteroids detected in herb, while none were found in callus. The ubiquitous presence of triterpenoid saponins in tested *L. flos-cuculi* extracts hints that they are the compounds responsible, in accordance with their role in plants as phytoalexins and natural antifungal agents [34]. It has been stated that medicinal plant species possessing antibacterial or antifungal properties can also exhibit antiprotozoal activity [17,35], especially if containing active constituents of a steroid or saponin structure that are able to permeabilize and destroy the cell membrane of a pathogenic microorganism. This prompted our team to investigate the antiprotozoal activity of fractionated extracts and isolated ecdysteroids (20E and polB).

It was previously mentioned that pharmacotherapy against *Acanthamoeba* infections is not always effective due to resistance, while the drugs used exert many adverse effects and toxicity. As the treatment requires combined therapy utilizing antibiotics, antifungal drugs, and disinfectants, it is difficult to choose a good candidate for the positive control in *Acanthamoeba* bioassays. There are data supporting the amoebicidal properties of polyene macrolides, such as nystatin or amphotericin B, in the concentration range of 10–100 µg/mL [36,37]. On that basis, nystatin was selected as a positive control and used in the concentration range of 50–200 µg/mL. Although it exerted a fairly high amoebicidal effect, it can be seen that the amoebae started to develop resistance on the third day (Figure 7).

The amoebicidal effect differed significantly between 40% and 80% methanolic fractions, as well as between the origin of the extract: Roots; herb; and callus. The 80% methanolic root fraction was the most potent, exerting a visibly strong amoebicidal effect at a 0.1 mg/mL concentration: Almost a 50% decrease of trophozoites by the first day and about 75% inhibition at day three. It exerted an even stronger effect at 0.5 mg/mL, reaching 75% inhibition at day one and nearly 90% at day three. None of the other assessed fractions exhibited such potency at concentrations below 1 mg/mL. In summary, the 80% methanolic fractions derived from extracts of studied plant material were generally more effective than 40% methanolic fractions, which can be attributed to their high triterpenoid saponin content. The 80% root fraction caused the strongest growth reduction of amoebae at a very low concentration of 0.05 mg/L, while both fractions of flowering herb extract were barely able to inhibit the growth by half at the highest concentrations tested. Interestingly, the efficacy of the 80% herb fraction peaked at the first day of incubation. Callus fractions exhibited moderate activity. Similarly, the isolated ecdysteroids were also moderately effective, requiring a concentration of 0.5 mg/mL and three days for almost complete growth reduction of *Acanthamoeba* trophozoites. Despite the promising activity of both fractions and compounds in many cases, the statistical significance of antiamoebic activity is limited to the highest concentrations of root and callus fractions, as well as crude extracts. Purified ecdysteroids exert a statistically significant effect at all concentrations, but only after the third day, so the effect is time-dependent. The herb fraction activity is so mediocre that it cannot be regarded as statistically significant in the range of concentrations tested.

Evaluations of the activity of other medicinal plants against the *Acanthamoeba castellani* trophozoite stage have been frequently reported in recent review publications [14,15,16,38]. The activity of *L. flos-cuculi,* compared to the amoebicidal activity of extracts from other plant species, is fairly high. For example, the effective concentration of an *Eryngium alpinum* shoot culture extract was 0.5 mg/mL, displaying a similarly high potency of an in vitro-derived material extract rich in triterpenoid saponins [39]. However, the comparison of IC_50_ values for extracts of plants especially effective against *Acanthamoeba*, including *Solidago virgaurea* flowers (0.01 mg/mL), *Solidago graminifolia* flowers (0.05 mg/mL), *Pueraria lobata* root (0.01 mg/mL), and *Rubus chamaemorus* leaves (0.05 mg/mL) [40], shows that only the 80% methanolic fraction of the *L. flos-cuculi* root extract exerts a similarly strong activity, with IC_50_ at 0.06 mg/mL. The isolated ecdysteroids are comparably strong, both with an IC_50_ at 0.07 mg/mL, but the IC_50_ values for all other fractions are higher. Studies on the effect of diverse fractions from *Eryngium planum* leaf and root extracts demonstrated a significant potency of saponin-containing fractions at low concentrations (1 mg/mL) [41]. Similarly, the extract from the leaves of *Chaenomeles japonica* shoot cultures, at a concentration of 1 mg/mL, inhibited the *Acanthamoeba* trophozoite growth by over 90% after three days of treatment. The effect was primarily attributed to pentacyclic triterpenoids and phenolic acids [42]. It was mentioned by Derda et al. [41], while comparing the amoebistatic activity of several fractions derived from *Eryngium planum*, that the flavonoid fraction from leaves actually stimulated the growth of *Acanthamoeba*, suggesting a protective response. Therefore, the presence of flavonoids may at least decrease the amoebicidal activity of a fraction. Our results for crude, unfractionated extracts showed an amoebistatic effect at concentrations of 5–10 mg/mL. Certain compounds present in tested extracts may actually exhibit antagonistic effects instead of synergism, as it seems that the fractionation of root extract constituents significantly increases the antiamoebic activity of both resulting fractions. It is possible that flavonoids in a herb fraction may actually exert cytoprotective activity, just as they affect multicellular organisms. However, callus fractions containing neither ecdysteroids nor flavonoids are still less toxic than root fractions against *Acanthamoeba.* This may be caused by the qualitative difference between saponin complexes in roots and callus, and as a result, different antiamoebic activity [18]. However, considering the significant amoebicidal activity of 20E and polB, the absence of flavonoids combined with the high content of ecdysteroids in the 80% root fraction might contribute to its exceptionally high amoebicidal activity, perhaps by a different mechanism of action.

Since 20E and polB revealed almost identical antiamoebic activity at the same concentrations at each day of treatment (Figure 6), the sum of both compounds in respective dilutions was compared to the observed activity against *Acanthamoeba* and is presented in Table S3 (Supplementary Material). No clear correlation was observed between the ecdysteroid concentration in samples and trophozoite growth inhibition. Ecdysteroid concentrations in herb and root fraction dilutions exhibit a much smaller range (1–30 µg/mL) than the concentrations of pure compounds (50–500 µg/mL). While this is sufficient for root fractions to be effective, pure ecdysteroids require a concentration of 100 µg/mL to inhibit *Acanthamoeba* growth by over 50% or an effective concentration of 500 µg/mL to reach >95% inhibition. The 80% root fraction is effective at very low dilutions (0.05–0.5 mg/mL), and while the dilution of 0.1 mg/mL contains only 6.175 µg/mL of ecdysteroid sum, it is able to inhibit the amoeba growth by 73.79%, which also points to the activity of other constituents. On the contrary, the crude herb extract at a dilution of 10 mg/mL contains over 250 µg/mL of ecdysteroids, but barely reached 50% inhibition. This hints that other constituents, e.g., the flavonoids mentioned before, might attenuate its activity. The same dilution of root extract is both very rich in ecdysteroids (375 µg/mL) and causes significant inhibition (91.10%). Finally, callus fractions exert significant antiamoebic activity, despite containing no ecdysteroids. This suggests that while ecdysteroids are partially responsible for the observed activity, the species contains other metabolites, most likely triterpenoid saponins, that contribute to the majority of the amoebicidal effect.

The Microtox assay is a quick and sensitive test for screening of the toxicity or antimicrobial activity of various samples, such as polluted water and soil samples [43] or bioactive substances at concentrations similar to those achieved in vivo [44]. It is based on bioluminescent *Aliivibrio fischeri* bacteria with a damaged cell wall to facilitate the exposure to bioactive agents. The results of the acute toxicity assessment using Microtox^®^ show that all of the tested materials differed in terms of the exhibited toxicity. The range of concentrations tested was the same as the range of concentrations used in antiamoebic evaluation. The only non-toxic solution was the polypodine B at a 0.5 mg/mL concentration, as the arbitrary threshold for non-toxic compounds is 20% [43]. This suggests that the 5-β-hydroxyl group present in polypodine B and not in 20-hydroxyecdysone might affect the biological activity of this compound, as 20-hydroxyecdysone exhibited an almost five times higher toxic effect in the Microtox^®^ test conditions. Interestingly, the toxicities obtained for the 40% methanolic extracts of the root, herb, and callus were far less toxic compared to the 80% fractions, even when tenfold higher concentrations were used. Such a phenomenon was also observed in the literature and may be attributed to the antibacterial properties exhibited by the substances present in the fractions extracted with less polar solvent mixtures [44]. *Aliivibrio fischeri* are gram negative bacteria and because of that, the toxicity testing might sometimes be influenced by the antimicrobial potential of the molecules tested [45]. This observation is in agreement with the TLC-detected constituents of 80% methanolic fractions of all tested materials, as they were especially abundant in triterpenoid saponins, known for their potent antimicrobial activity [34]. Similarly, the toxicity exhibited by purified ecdysteroids supports the observations of Mamadalieva et al. [11] in terms of their antibacterial effect, although the content of ecdysteroids in fraction dilutions (Table S2, Supplementary Material) seems like it does not affect the observed toxicity in this model. It is important to note that the acute toxicity indicated by the Microtox^®^ results does not imply the same toxicity in mammals. The LD_50_ of 20-hydroxyecdysone in mammals is actually very low, at 6 g/kg of body mass in a murine model [9], while the effect exerted by 80% methanolic fractions is most likely the antimicrobial effect of their saponin constituents. Therefore, the results do not exclude the potential therapeutic use of *L. flos-cuculi* preparations.

## 4. Materials and Methods

### 4.1. Plant Material

The plants of *Lychnis flos-cuculi* were gathered in June 2016 from a meadow near Kuźnica Trzcińska, Wielkopolskie Voivodeship, Poland (51°09′21′′ N 18°03′24′′ E), together with mature seeds that were used for plant propagation in vitro through axillary bud formation, according to our previously described protocol [6]. Micropropagated plants were transferred to soil in an experimental plot, where they reached the stage of flowering. The voucher specimens (No. CP-Lfc-2016-0601) were deposited in the Herbarium of Department of Pharmaceutical Botany and Plant Biotechnology of Poznan University of Medical Sciences. The studied callus was induced from hypocotyl of axenic seedlings, as described by Maliński et al. [6]. The flowering herb and roots of micropropagated plants were harvested from the experimental plot in June 2018, three months after the transfer to soil, and along with callus used for the preparation of extracts to evaluate their antifungal and antiamoebic activity, and acute toxicity. For the isolation of ecdysteroids, the flowering herb of micropropagated plants was selected, given its highest content of these compounds.

### 4.2. Extraction and Fractionation

The flowering herb and roots of *L. flos-cuculi* from in vitro-derived plants were harvested from the experimental plot, dried, and pulverized. The stabilized callus, after six passages, was collected and lyophilized.

For a preliminary evaluation of the antifungal activity, methanolic extracts were prepared by triple maceration under reflux, at 85 °C. For other purposes, all plant materials were extracted three times with 80% methanol under reflux, at 85 °C. The crude extracts were evaporated to dryness and redissolved in water. Solid phase extraction was used to prepare the fractions. Each extract was dissolved in water (target concentration of 0.2 g/mL) and fractionated using SepPak (Waters) RP-18 microcolumns. Every 1 mL of aqueous solution of the extract was first eluted with 5 mL of water and 5 mL of 40% aqueous methanol, combined to yield a 40% methanolic fraction, and then with 5 mL of aqueous 80% methanol and 5 mL of pure methanol, combined to yield an 80% methanolic fraction. The procedure was repeated for each extract, resulting in two fractions—40% and 80% methanolic fractions—for all plant material. The fractions were evaporated to dryness and used for further experiments.

### 4.3. Isolation of 20-hydroxyecdysone (**1**) and Polypodine B (**2**)

The flowering herb of micropropagated *L. flos-cuculi* was gathered, dried, and coarsely ground. The base extract was prepared by the exhaustive percolation of 80.0 g of dry plant material with methanol (POCh, Gliwice, Poland) used as an extractant. The percolate was repeatedly decanted into a large round-bottom flask and dried using a rotary evaporator, and the condensed methanol was reused as an extractant. A total of 7 L of methanol was used for percolation.

The resulting dry extract was suspended in distilled water and exhaustively extracted with dichloromethane (ChemPur, Piekary Śląskie, Poland) until only traces of chlorophyll were visible in the organic phase. The dichloromethane fraction was discarded, and the aqueous fraction (I) was evaporated to dryness, before being dissolved in distilled water. The solution was exhaustively extracted with *n*-butanol (ChemPur, Piekary Śląskie, Poland) saturated with water, yielding an aqueous fraction (II) and butanolic fraction. The aqueous fraction (II) was discarded and the dried butanolic fraction was used for the next steps.

The butanolic fraction was dissolved in methanol (target concentration of 0.3 g/mL) with the aid of an ultrasonic bath. The resulting suspension was allowed to sediment and the supernatant was used. Because of the excellent solubility of ecdysteroids in methanol, the precipitate was discarded as it was unlikely it would contain them.

The supernatant was manually deposited as a continuous line on the bottom of 20 × 20 cm^2^ silica gel glass plates (Merck) with a 254 nm fluorescence indicator. The plates were developed in dichloromethane-methanol (15:2, *v*/*v*), 4–6 times, depending on marginal differences in the amount of the deposited fraction, and how many ecdysteroids were separated from one another and the rest of the constituents. Between developments, the position of ecdysteroids was monitored under UV 254 nm light, which is a wavelength very close to the absorption maximum (242 nm) of the ecdysteroid cholest-7-en-6-one backbone. When the R*f* values of both compounds reached ca. 0.4–0.5, with the difference between them being at least 0.05, the preparative TLC was finished. Two separate batches of silica gel, each one containing one of the compounds, were collected manually from every plate. Using a Buchner funnel attached to the vacuum pump, each batch of the silica gel was rinsed with a 1:1 (*v*/*v*) dichloromethane-methanol mixture. The resulting eluates were evaporated to dryness, yielding crude compounds with a yellowish tint.

Despite the high efficacy of the preparative TLC approach, at least one compound of the flavonoid structure was co-eluted with each ecdysteroid. To purify the crude isolates, they were dissolved in distilled water and subjected to solid phase extraction on RP-18 SepPak (Waters) cartridges. Each sample was eluted with 5 mL volumes of water and 20%, 40% (three times), 80%, and 100% methanol, yielding separate subfractions which were then evaporated to dryness and inspected by TLC. Aqueous, 20%, 80%, and methanolic subfractions contained impurities and were discarded.

The 40% aqueous methanolic subfractions of both compounds were evaporated, redissolved in HPLC-grade methanol (POCh, Gliwice, Poland), and ultimately purified in a 10 cm long chromatographic column, filled with neutral aluminum oxide (50–150 µm, Fluka) suspended in water and equilibrated with methanol. Each sample was eluted with ca. 40 mL of methanol. The eluate was evaporated to dryness, yielding white crystalline residue. The crystallines **1** (349 mg, 0.437% final yield) and **2** (271 mg, 0.339% final yield) were then used for further experiments and NMR structure elucidation.

### 4.4. Chemical Structure Confirmation

NMR spectra were recorded on an Avance III Bruker (500 MHz) spectrometer (Bruker, Billerica, MA, USA) in CD_3_OD using solvent residual signals at 3.31 ppm for ^1^H and at 49.05 ppm for ^13^C as an internal shift reference. Fourier transform infrared (FT-IR) spectra were recorded on a Bruker IR spectrometer, in the range of 400–4000 cm^−1^, with KBr as a blank. UV-Vis spectra were recorded on a Hitachi UV/VIS U-1900 spectrophotometer (Hitachi Ltd., Tokyo, Japan). High resolution mass spectra (MALDI) were recorded on AB Sciex LC/MS/MS System API 4000 QTRAP at the Department of Inorganic and Analytical Chemistry at Poznan University of Medical Sciences. High-performance liquid chromatography analyses were performed on Agilent 1200 SL HPLC (Agilent Technologies, Santa Clara, CA, USA) equipped with a diode array detector set at 242 nm. The conditions of the separation were the same as described in our previous study by Maliński et al. [6].

#### 4.4.1. Compound Characterization

##### Compound **1** (20-hydroxyecdysone)

White powder. UV-Vis (ethanol); λmax nm (logε): 242 (4.07). HR-MS (MALDI) *m*/*z* found: 519.2788, [M + K]^+^ C_27_H_44_O_7_K requires 519.2719. FT-IR (KBr, ν_max_/cm^−1^): 2958s (CH3, CH2), 2927s (CH3, CH2), 2872s (CH3, CH2), 1678s (C=O) 1647s (C=C), 1635s (C=C), 1558s, 1379s (CH3, C-OH), 1348s (C-OH), 1313w (C-OH), 1261w (C-OH), 1224w (CH3, C-OH), 1141s (C-CH3), 1114w (C-C), 1051s (cyclohexane, CH2-OH), 1022w (cyclohexane), 993w (C-C), 950w (C-H), 875s, 840w (C-C). ^1^H and ^13^C NMR: See Table 1.

##### Compound **2** (polypodine B)

White powder. UV-Vis (ethanol); λmax nm (logε): 237 (4.03). HR-MS (MALDI) *m*/*z* found: 519.2921, [M + Na]^+^ C_27_H_44_O_8_Na requires 519.2928. FT-IR (KBr, ν_max_/cm^−1^): 2933s (CH3, CH2), 2918s (CH3, CH2), 2848s (CH3, CH2), 1683s (C=O), 1652s (C=C), 1635s (C=C), 1558s, 1346s (C-OH), 1338w (C-OH), 1307w (C-OH), 1302w (C-OH), 1286w (C-OH), 1271w (C-OH), 1253w (C-OH), 1223w (CH3, C-OH), 1136s (C-CH3), 1070s (cyclohexane, CH2-OH), 1053s (cyclohexane, CH2-OH), 1012w (cyclohexane), 993w (C-C), 877s, 842w (C-C). ^1^H and ^13^C NMR: See Table 1.

Additional data are provided in Supplementary Material (Figures S1–S19).

### 4.5. Antifungal Activity

Extracts of flowering herb, roots, and callus prepared with 100% methanol were used for antifungal activity assays. The species of fungi used in this study were *Candida albicans* ATCC 10231, *Cryptococcus neoformans* clinical strain, *Trichophyton mentagrophytes* ATCC 9533, *T. rubrum* ATCC 28188, *Aspergillus brasiliensis* ATCC 16404, and *A. fumigatus* ATCC 204305.

The minimal inhibitory concentration (MIC mg/mL) values for extracts of *L. flos-cuculi* were determined according to the European Society of Clinical Microbiology and Infectious Diseases (EUCAST) recommendations, using the serial microdilution method on polystyrene plates with RPMI-1640-*L*-glutamine (without sodium bicarbonate) (Sigma-Aldrich, St. Louis, MO, USA)) as a medium. Concentrated solutions of *L. flos-cuculi* extracts were dissolved in RPMI-1640 to obtain the required concentration. To each well of polystyrene plates, 100 μL of the appropriate pre-prepared concentrations of tested extracts was applied. Then, 100 μL of final inoculum of all studied organisms (about 5 × 10^5^ CFU/mL (Colony Forming Units per mL)) was added. The plates were incubated at 35 °C for 24–48 h. After the appropriate incubation time, the presence (or absence) of growth was observed visually. The MIC was defined as the lowest sample concentration that produced visible inhibition of fungal growth. Additionally, microbial growth and broth control was conducted. All tests were carried out according to the same instructions for each strain, three times for each extract. Nystatin was used as a positive control.

### 4.6. Antiamoebic Activity Assay

In this study, the *Acanthamoeba* sp. strain Ac55 (isolated from a patient with keratitis, T4 genotype) deposited in GenBank (NCBI) under accession number KP120880 was used. The amoebae were axenically cultured on a liquid medium containing 2% Bacto-Casitone. 

The fractions of 80% aqueous methanolic extracts and purified compounds were dissolved in 50 µL of dimethylsulfoxide (DMSO) (Sigma-Aldrich, St. Louis, MO, USA) and then diluted with distilled water to obtain the appropriate concentrations. These dilutions were added to the axenic culture of amoebae containing 5 × 10^4^ cells/mL at the concentrations of 0.05–5 mg/mL. The increase or decrease in the number of amoebae was checked at 24-h intervals during three days in a Thoma hemocytometer chamber. The control consisted of cultured trophozoites without fractions. The relationship between the fraction concentration and the time of treatment of trophozoite cultures was investigated. Nystatin was selected as a positive control at concentrations ranging from 0.05 to 0.2 mg/mL.

### 4.7. Evaluation of Acute Toxicity Using the Microtox^®^ Acute Toxicity Test

The acute toxicity of the compounds/extracts was tested using the Microtox^®^ acute toxicity test—81.9% Screening Test—which was performed using Microtox^®^ M500 equipment according to the protocols distributed by the producer (ModernWater plc) [46,47]. Cell viability was calculated according to bioluminescence emitted by the *Aliivibrio fischeri* bacteria, as measured with Microtox^®^ M500 with Modern Water MicrotoxOmni 4.2 software. Appropriate concentrations (mg/mL) of the compounds/extracts were prepared using distilled water.

### 4.8. Evaluation of the Ecdysteroid Content in Studied Fractions

The quantitative chromatography analyses were performed on Agilent 1200 SL HPLC equipped with a diode array detector. Analytical separation and guard columns were RP Select B Lichrospher 60, LiChroCART 125–4 5 μm (Merck, Darmstadt, Germany). All of the experiments were carried out as described in our previous work [6]. The UV absorption was measured at λ_max_ = 242 nm in methanol. The chromatography step was conducted using gradient elution of mobile phase consisting of methanol and water, according to the following scheme: t_0_ [min]—5% MeOH; t_10_—30% MeOH; t_27_—30% MeOH; and t_32_—5% MeOH. The samples were dissolved in methanol, at a concentration of 10 mg/mL.

### 4.9. Statistical Analysis

Statistica 13 (StatSoft, Inc., Tulsa, OK, USA) software was used for performing the statistical analyses. The gathered data were subjected to a one-way analysis of variance (ANOVA), as well as Duncan’s post hoc test. To determine statistical significance, a two-sided *p* value of 0.05 was applied.

## 5. Conclusions

The implemented method of isolation used is a relatively quick, efficient purification process which can be an alternative to work and resource-consuming methods of ecdysteroid isolation based almost entirely on column chromatography. It allows real-time observation of the ecdysteroid position on a plate which is non-destructive to the sample. The method can be modified and adjusted to focus on other ecdysteroid compounds with diverse structures. The flowering herb of micropropagated plants of *Lychnis flos-cuculi* is a reliable, rich source of 20-hydroxyecdysone and polypodine B.

The study indicated that *L. flos-cuculi* root and callus extracts possess significant antiamoebic activities. The roots are easily obtainable and can be considered a promising source of bioactive compounds with amoebicidal action against *Acanthamoeba* trophozoites. The complex of triterpenoid saponins present in root and callus extracts are, most probably, responsible for the amoebicidal effect. The acute toxicity observed is most likely the result of the strong antibacterial activity against *Aliivibro fischeri* bacteria, but does not exclude the use of fractions or compounds as therapeutic agents.

To the best of our knowledge, this is the first mention of amoebicidal activity of this species. *L. flos-cuculi* plant material obtained by in vitro clonal propagation is characterized by genetic uniformity and phytochemical homogeneity. Extracts derived from this material can be considered novel natural agents with therapeutic potential against *Acanthamoeba* trophozoites, in concentrations that are not toxic. Further research is needed to elucidate the structure of the most active ingredients, and assess the efficacy and safety of *L. flos-cuculi* preparations, at effective concentrations in an in vivo model.

## Figures and Tables

**Figure 1:**
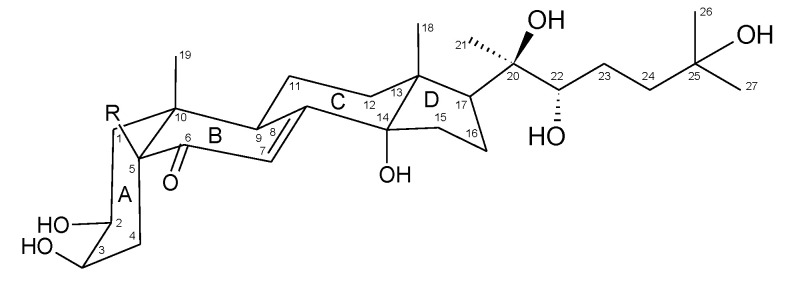
Chemical structure of 20-hydroxyecdysone (R=H, compound **1**) and polypodine B (R=OH, compound **2**), the major ecdysteroid constituents of *Lychnis flos-cuculi.*

**Table 1:** NMR spectral data of compounds **1** and **2** (in CD_3_OD).

	20-Hydroxyecdysone (1)		Polypodine B (2)	
Carbon	^13^C [ppm]	^1^H [ppm (Hz)]	^13^C [ppm]	^1^H [ppm (Hz)]
1	37.4	1.78 m 1.42 m	34.2	1.73 m 1.70 m
2	68.7	3.83 ddd (11.9, 4.4, 3.1)	68.4	3.94 ddd (10.3, 6.5, 3.1)
3	68.5	3.94 ddd (3.1, 3.0, 3.0)	70.3	3.98 ddd (3.1, 3.1, 3.1)
4	32.9	1.72 m 1.69 m	36.2	2.07 dd (14.7, 3.1) 1.76 m
5	51.8	2.37 dd (12.9, 4.5)	80.3	-
6	206.5	-	202.4	-
7	122.2	5.80 d (2.6)	120.6	5.84 d (2.6)
8	168.0	-	167.6	-
9	35.1	3.15 ddd (10.5, 7.1, 2.6)	39.0	3.18 ddd (10.8, 7.2, 2.6)
10	39.3	-	45.5	-
11	21.6	1.81 m 1.70 m	22.5	1.79 m 1.75 m
12	32.6	2.12 ddd (13.0, 13.0, 5.0) 1.88 m	32.6	2.13 ddd (13.0, 13.0, 5.1) 1.87 m
13	48.7	-	48.7	-
14	85.3	-	85.1	-
15	31.8	1.70 m 1.58 m	31.8	1.96 m 1.57 m
16	21.5	1.98 m 1.80 m	21.5	1.98 m 1.73 m
17	50.6	2.38 dd (9.8, 8.3)	50.5	2.38 dd (9.4, 8.4)
18	18.1	0.88 s (3H)	18.1	0.88 s (3H)
19	24.5	0.96 s (3H)	17.0	0.91 s (3H)
20	77.9	-	77.9	-
21	21.1	1.19 s (3H)	21.1	1.196 s (3H)
22	78.5	3.33 dd (10.4, 1.8)	78.5	3.32 dd (10.1, 1.5)
23	27.4	1.65 m 1.28 m	27.4	1.65 m 1.27 m
24	42.4	1.79 m 1.42 m	42.4	1.80 m 1.42 m
25	71.3	-	71.3	-
26	29.0	1.18 s (3H)	29.0	1.191 s (3H)
27	29.8	1.20 s (3H)	29.8	1.204 s (3H)

The symbols s, d, dd, ddd, and m stand for singlet, doublet, doublet of doublets, doublet of doublet of doublets, and multiplet, respectively.

**Table 2:** Minimum inhibitory concentration (MIC, mg/mL) values of methanolic extracts from the callus, flowering herb, and roots of micropropagated *Lychnis flos-cuculi* tested against pathogenic fungi. Nystatin was used as a positive control.

Sample	Minimum Inhibitory Concentration [mg/mL]					
	*Candida albicans ^a^*	*Cryptococcus neoformans ^a^*	*Trichophyton mentagrophytes ^b^*	*Trichophyton rubrum ^b^*	*Aspergillus brasiliensis ^c^*	*Aspergillus fumigatus ^c^*
Callus	2.5	1.25	2.5	2.5	2.5	2.5
Herb	2.5	2.5	2.5	2.5	2.5	2.5
Roots	2.5	1.25	2.5	2.5	1.25	2.5
Nystatin	0.004	0.004	0.004	0.004	0.004	0.004

Opportunistic yeasts ^a^, dermatophytes ^b^, and molds causing pulmonary aspergillosis ^c^.

**Table 3:** The effect of the crude 80% aqueous methanolic extracts from the herb and roots of *Lychnis flos-cuculi* at different concentrations on *Acanthamoeba* trophozoite inhibition during five-day treatment.

	Extract	Duration of Treatment [Days]		
Extract	Concentration	1st Day	2nd Day	5th Day
	[mg/mL]	MT ± SD	MT ± SD	MT ± SD
Herb	control	10.17 ± 3.53	24.25 ± 6.71	35.58 ± 7.45
	1	10.11 ± 2.02	23.25 ± 8.14	28.75 ± 7.55
	5	9.83 ± 3.36	21.92 ± 10.16	26.25 ± 7.85
	10	5.00 ± 1.63	13.33 ± 2.62 *	18.83 ± 3.97 *
Roots	control	10.17 ± 3.53	24.25 ± 6.71	35.58 ± 7.45
	1	8.58 ± 3.35	18.23 ± 3.38	24.58 ± 13.69
	5	7.67 ± 2.60	13.33 ± 2.13 *	18.83 ± 10.56
	10	4.17 ± 1.52 *	4.00 ± 3.14 *	3.17± 2.76 *

MT, mean trophozoite number in the hemocytometer chamber. * *p* < 0.05 statistically significant difference in comparison with the control during the same time interval; number of replicates *n* = 18.

**Table 4:** The effect of the 40% and 80% aqueous methanolic fractions of the extract from roots of micropropagated *Lychnis flos-cuculi* at different concentrations on *Acanthamoeba* trophozoite inhibition during three-day treatment.

Fraction	Fraction	Duration of Treatment [Days]		
	Concentration	1st Day	2nd Day	3rd Day
	[mg/mL]	MT ± SD	MT ± SD	MT ± SD
40% aqueous	control	6.13 ± 1.96	20.65 ± 3.79	33.00 ± 3.61
methanolic fraction	0.5	5.58 ± 3.35	18.23 ± 3.38	24.58 ± 13.69
	2.5	4.67 ± 2.60	13.33 ± 2.13	18.83 ± 10.56
	5	4.17 ± 1.52	4.00 ± 3.14 *	4.17 ± 2.76 *
80% aqueous	control	8.89 ± 2.00	17.89 ± 2.40	23.50 ± 5.24
methanolic fraction	0.05	8.17 ± 1.83	15.33 ± 4.57	18.91 ± 8.73
	0.1	4.61 ± 1.89 *	5.18 ± 3.24 *	6.16 ± 1.57 *
	0.5	3.13 ± 1.03 *	2.83 ± 1.95 *	2.67 ± 2.01 *

MT, mean trophozoite number in the hemocytometer chamber. * *p* < 0.05 statistically significant difference in comparison with the control during the same time interval; number of replicates *n* = 18.

**Table 5:** The effect of the 40% and 80% aqueous methanolic fractions of the extract from the flowering herb of micropropagated *Lychnis flos-cuculi*, at different concentrations, on *Acanthamoeba* trophozoite inhibition during three-day treatment.

Fraction	Fraction	Duration of Treatment [Days]		
	Concentration	1st Day	2nd Day	3rd Day
	[mg/mL]	MT ± SD	MT ± SD	MT ± SD
40% aqueous	control	5.28 ± 0.80	10.06 ± 3.95	19.17 ± 3.37
methanolic fraction	0.1	4.89 ± 2.50	9.21 ± 3.27	17.06 ± 4.13
	0.5	4.63 ± 1.41	9.00 ± 3.31	16.72 ± 3.18
	1	4.60 ± 1.80	8.25 ± 2.05	15.72 ± 2.13
80% aqueous	control	5.28 ± 0.80	10.06 ± 3.95	19.17 ± 3.37
methanolic fraction	0.1	3.74 ± 1.73	8.71 ± 3.27	16.56 ± 4.02
	0.5	3.27 ± 2.80	7.50 ± 3.76	13.71 ± 5.58
	1	3.11 ± 1.97	7.01 ± 3.27	12.44 ± 6.22

MT, mean trophozoite number in the hemocytometer chamber; number of replicates *n* = 18.

**Table 6:** The effect of the 40% and 80% aqueous methanolic fractions of the *Lychnis flos-cuculi* callus extract, at different concentrations, on *Acanthamoeba* trophozoite inhibition during three-day treatment.

Fraction	Fraction	Duration of Treatment [Days]		
	Concentration	1st Day	2nd Day	3rd Day
	[mg/mL]	MT ± SD	MT ± SD	MT ± SD
40% aqueous	control	8.89 ± 2.00	17.89 ± 2.40	23.50 ± 5.24
methanolic fraction	0.5	6.22 ± 2.24	12.26 ± 3.46	14.11 ± 5.85
	1	4.65 ± 1.68 *	7.13 ± 2.43 *	6.41 ± 4.04 *
	5	1.44 ± 1.80 *	2.18 ± 1.80 *	2.05 ± 1.98 *
80% aqueous	control	6.13 ± 1.96	20.65 ± 3.79	33.00 ± 3.61
methanolic fraction	0.2	5.58 ± 3.35	18.23 ± 3.38	24.58 ± 13.69
	1	4.67 ± 2.60	13.33 ± 2.13 *	18.83 ± 10.56
	2	4.17 ± 1.52	4.00 ± 3.14 *	3.17 ± 2.76 *

MT, mean trophozoite number in the hemocytometer chamber. * *p* < 0.05 statistically significant difference in comparison with the control during the same time interval; number of replicates *n* = 18.

**Table 7:** The effect of 20-hydroxyecdysone and polypodine B isolated from *Lychnis flos-cuculi* on *Acanthamoeba* trophozoite inhibition during three-day treatment.

Compound	Sample	Duration of Treatment [Days]		
	Concentration	1st Day	2nd Day	3rd Day
	[mg/mL]	MT ± SD	MT ± SD	MT ± SD
20-hydroxyecdysone	control	8.14 ± 1.96	21.65 ± 2.99	35.18 ± 3.55
(**1**)	0.05	6.98 ± 2.33	16.96 ± 3.11	20.50 ± 5.69 *
	0.1	6.09 ± 2.67	12.90 ± 2.22 *	13.17 ± 2.76 *
	0.5	4.89 ± 1.25 *	6.15 ± 1.16 *	0.74 ± 0.46 *
Polypodine B	control	8.14 ± 1.96	21.65 ± 2.99	35.18 ± 3.55
(**2**)	0.05	7.16 ± 3.33	17.87 ± 3.11	22.47 ± 5.69 *
	0.1	6.21 ± 2.67	13.90 ± 2.22 *	15.16 ± 2.76 *
	0.5	4.23 ± 1.25 *	4.01 ± 1.16 *	0.96 ± 0.76 *

MT, mean trophozoite number in the hemocytometer chamber. * *p* < 0.05 statistically significant difference in comparison with the control during the same time interval; number of replicates *n* = 12.

**Table 8:** Determination of *Acanthamoeba* trophozoite IC_50_ (mg/mL) for the studied 40% and 80% aqueous methanolic fractions of *Lychnis flos-cuculi* extracts and isolated ecdysteroids.

Sample		IC_50_ [mg/mL]	
	1st Day	2nd Day	3rd Day
Root, 40% fraction	>5.00	3.30	2.95
Root, 80% fraction	>0.50	0.06	0.06
Herb, 40% fraction	>1.00	>1.00	>1.00
Herb, 80% fraction	>1.00	>1.00	>1.00
Callus, 40% fraction	1.30	0.70	0.55
Callus, 80% fraction	>2.00	1.35	1.15
20-hydroxyecdysone (**1**)	>0.50	0.13	0.07
Polypodine B (**2**)	>0.50	0.13	0.07

**Table 9:** The content of 20-hydroxyecdysone and polypodine B in fractions and crude extracts used for bioassays, expressed as the mg/g of dry weight of the sample.

Sample	Ecdysteroid Content [mg/g d.w.]		
	20-hydroxyecdysone	Polypodine B	Sum
Root, 40% fraction	2.99	2.45	5.44
Root, 80% fraction	36.87	24.88	61.75
Herb, 40% fraction	5.09	4.53	9.62
Herb, 80% fraction	18.26	11.44	29.7
Callus, 40% fraction	Not detected	Not detected	N/A
Callus, 80% fraction	Not detected	Not detected	N/A
Crude root extract	20.10	17.38	37.48
Crude herb extract	13.89	11.18	25.07

## Data Availability

The data presented in this study are available in Supplementary Material.

## Supplementary Materials

The following are available online. Figure S1: ^1^H NMR spectrum (500 MHz, CD_3_OD) of compound 1, Figure S2: ^13^C NMR spectrum (125 MHz, CD_3_OD) of compound 1, Figure S3: HH-COSY spectrum (500 MHz, CD_3_OD) of compound 1, Figure S4: HSQC spectrum (500/125 MHz, CD_3_OD) of compound 1, Figure S5: HMBC spectrum (500/125 MHz, CD_3_OD) of compound 1, Figure S6: NOESY spectrum (500 MHz, CD_3_OD) of compound 1, Figure S7: ^1^H NMR spectrum (500 MHz, CD_3_OD) of compound 2, Figure S8: ^13^C NMR spectrum (125 MHz, CD_3_OD) of compound 2, Figure S9: HH-COSY spectrum (500 MHz, CD_3_OD) of compound 2, Figure S10: HSQC spectrum (500/125 MHz, CD_3_OD) of compound 2, Figure S11: HMBC spectrum (500/125 MHz, CD_3_OD) of compound 2, Figure S12: NOESY spectrum (500 MHz, CD_3_OD) of compound 2, Figure S13. MALDI mass spectrum of 20-hydroxyecdysone (1), Figure S14: MALDI mass spectrum of polypodine B (2), Figure S15: Concentration-dependent UV-Vis spectra of 20-hydroxyecdysone recorded in ethanol, Figure S16: Concentration-dependent UV-Vis spectra of polypodine B recorded in ethanol, Figure S17: FT-IR spectra of 20-hydroxyecdysone (1) and polypodine B (2), Figure S18: HPLC-DAD chromatogram of isolated 20-hydroxyecdysone (1), Figure S19: HPLC-DAD chromatogram of isolated polypodine B (2), Figure S20: HPLC-DAD chromatogram of the 40% methanolic root fraction showing 20-hydroxyecdysone and polypodine B, Figure S21: HPLC-DAD chromatogram of the 80% methanolic root fraction showing 20-hydroxyecdysone and polypodine B, Figure S22: HPLC-DAD chromatogram of the 40% methanolic herb fraction showing 20-hydroxyecdysone and polypodine B, Figure S23: HPLC-DAD chromatogram of the 80% methanolic herb fraction showing 20-hydroxyecdysone and polypodine B, Figure S24: HPLC-DAD chromatogram of the 40% methanolic callus fraction showing no ecdysteroids, Figure S25: HPLC-DAD chromatogram of the 80% methanolic callus fraction showing no ecdysteroids, Table S1: The effect of nystatin (positive control) on *Acanthamoeba* trophozoite inhibition during three-day treatment, Table S2: The toxicity of studied 40% and 80% aqueous methanolic fractions from *Lychnis flos-cuculi* extracts and isolated ecdysteroids against *Aliivibrio fischeri* after 5 and 15 min, measured with the use of the Microtox assay, Table S3: Concentration of 20-hydroxyecdysone and polypodine B in respective dilutions of different *Lychnis flos-cuculi* material samples used in the antiamoebic activity bioassay.

## Sample Availability

Samples of the compounds are not available from the authors.
