# Supplementary material for: Two Ecdysteroids Isolated from Micropropagated Lychnis flos-cuculi and the Biological Activity of Plant Material
Source: Molecules. 2021 Feb 9;26(4):904. doi: 10.3390/molecules26040904 (PMC7914685; doi:10.3390/molecules26040904)
Supplement: Supplementary file 1 [file molecules-26-00904-s001.zip › molecules-1019812-supplementary-revised.pdf]

Supplementary Material

Article

Two Ecdysteroids Isolated from Micropropagated *Lychnis flos-cuculi* and Biological Activity of Plant Material

Michał P. Maliński [1]*, Jaromir Budzianowski [1], Małgorzata Kikowska [1], Monika Derda [2], Marcelina M. Jaworska [3], Dariusz T. Mlynarczyk [4] , Marta Szukalska [5], Ewa Florek [5] and Barbara Thiem [1]

[1] Chair and Department of Pharmaceutical Botany and Plant Biotechnology, Poznan University of Medical Sciences; 14 Sw. Marii Magdaleny St., 61-861 Poznań, Poland; jbudzian@ump.edu.pl (J.B.); kikowska@ump.edu.pl (M.K.); bthiem@ump.edu.pl (B.T.)
[2] Chair and Department of Biology and Medical Parasitology, Poznan University of Medical Sciences; 10 Fredry St., 61-701 Poznań, Poland; mderda@ump.edu.pl (M.D.)
[3] Chair and Department of Genetics and Pharmaceutical Microbiology, Poznan University of Medical Sciences; 4 Święcickiego St., 60-781 Poznań, Poland; marcelinajaworska@ump.edu.pl (M.J.)
[4] Chair and Department of Chemical Technology of Drugs, Poznan University of Medical Sciences, 6 Grunwaldzka St., 60-780 Poznań, Poland; mlynarczykd@ump.edu.pl (D.T.M.)
[5] Laboratory of Environmental Research, Department of Toxicology, Poznan University of Medical Sciences, 30 Dojazd St., 60-631 Poznań, Poland; martan@ump.edu.pl (M.N.); eflorek@ump.edu.pl (E.F.)
* Correspondence: mpmalinski@ump.edu.pl

Contents

Compound 1 (20-hydroxyecdysone)

Figure S1. ^{1}H NMR spectrum (500 MHz, CD_3OD) of compound **1.**

Figure S2. ^{13}C NMR spectrum (125 MHz, CD_3OD) of compound **1.**

Figure S3. HH-COSY spectrum of compound **1.**

Figure S4. HSQC spectrum (500/125 MHz, CD_3OD) of compound **1.**

Figure S5. HMBC spectrum (500/125 MHz, CD_3OD) of compound **1.**

Figure S6. NOESY spectrum (500 MHz, CD_3OD) of compound **2.**

Compound 2 (polypodine B)

Figure S7. ^{1}H NMR spectrum (500 MHz, CD_3OD) of compound **2.**

Figure S8. ^{13}C NMR spectrum (125 MHz, CD_3OD) of compound **2.**

Figure S9. HH-COSY spectrum (500 MHz, CD_3OD) of compound **2.**

Figure S10. HSQC spectrum (500/125 MHz, CD_3OD) of compound **2.**

Figure S11. HMBC spectrum (500/125 MHz, CD_3OD) of compound **2.**

Figure S12. NOESY spectrum (500 MHz, CD_3OD) of compound **2.**

Figure S13. MALDI mass spectrum of 20-hydroxyecdysone (**1**).

Figure S14. MALDI mass spectrum of polypodine B (**2**).

Figure S15. Concentration-dependent UV-Vis spectra of 20-hydroxyecdysone recorded in methanol.

Figure S16. Concentration-dependent UV-Vis spectra of polypodine B recorded in ethanol.

Figure S17. FT-IR spectra of 20-hydroxyecdysone (**1**) and polypodine B (**2**).

Figure S18. HPLC-DAD chromatogram of isolated 20-hydroxyecdysone (**1**).

Figure S19. HPLC-DAD chromatogram of isolated polypodine B (**2**).

Figure S20. HPLC-DAD chromatogram of 40% methanolic root fraction showing 20-hydroxyecdysone and polypodine B.

Figure S21. HPLC-DAD chromatogram of 80% methanolic root fraction showing 20-hydroxyecdysone and polypodine B.

Figure S22. HPLC-DAD chromatogram of 40% methanolic herb fraction showing 20-hydroxyecdysone and polypodine B.

Figure S23. HPLC-DAD chromatogram of 80% methanolic herb fraction showing 20-hydroxyecdysone and polypodine B.

Figure S24. HPLC-DAD chromatogram of 40% methanolic callus fraction showing no ecdysteroids.

Figure S25. HPLC-DAD chromatogram of 80% methanolic callus fraction showing no ecdysteroids.

Table S1. The effect of nystatin (positive control) on *Acanthamoeba* trophozoite inhibition during three-day treatment.

Table S2. The toxicity of studied 40% and 80% aqueous methanolic fractions from *Lychnis flos-cuculi* extracts and isolated ecdysteroids against *Aliivibrio fischeri* after 5 and 15 minutes, measured with the use of Microtox assay.

Table S3. Concentration of 20-hydroxyecdysone and polypodine B in respective dilutions of different *Lychnis flos-cuculi* material samples used in antiamoebic activity bioassay.

Compound 1 (20-hydroxyecdysone)

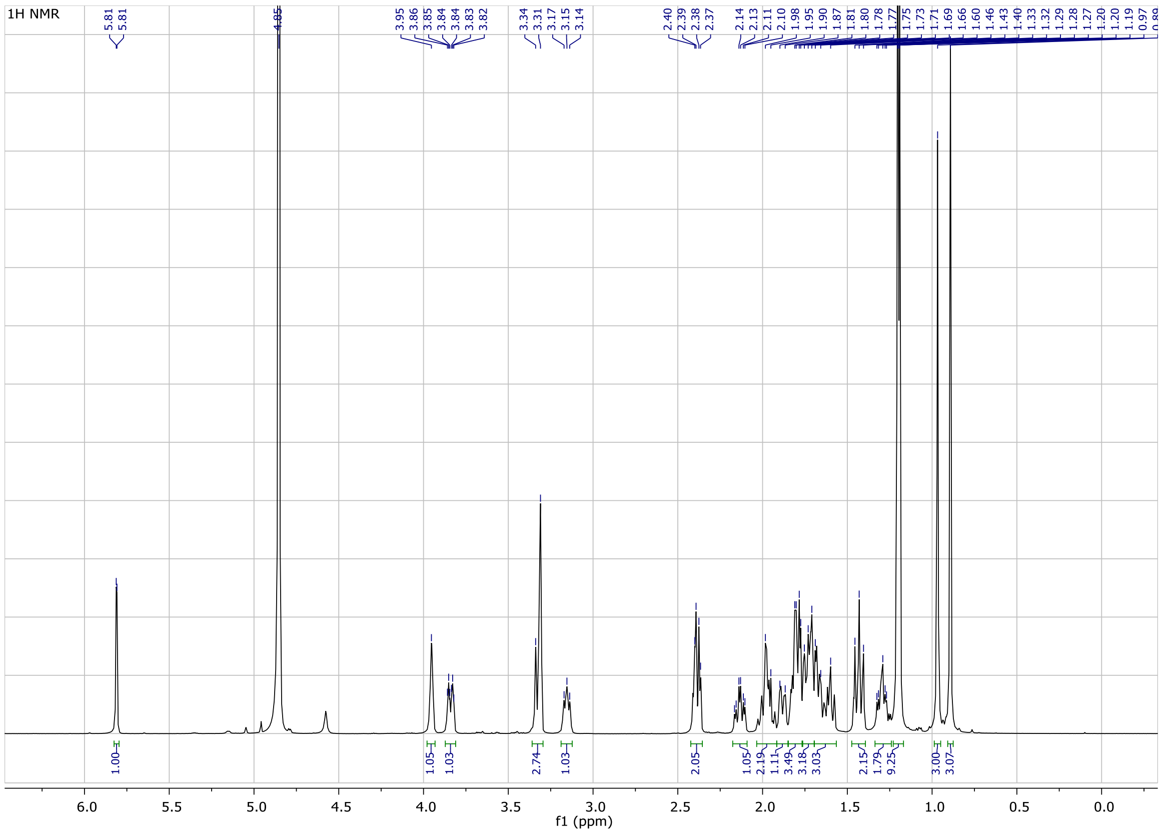

Figure S1. ^{1}H NMR spectrum (500 MHz, CD_3OD) of compound **1.**

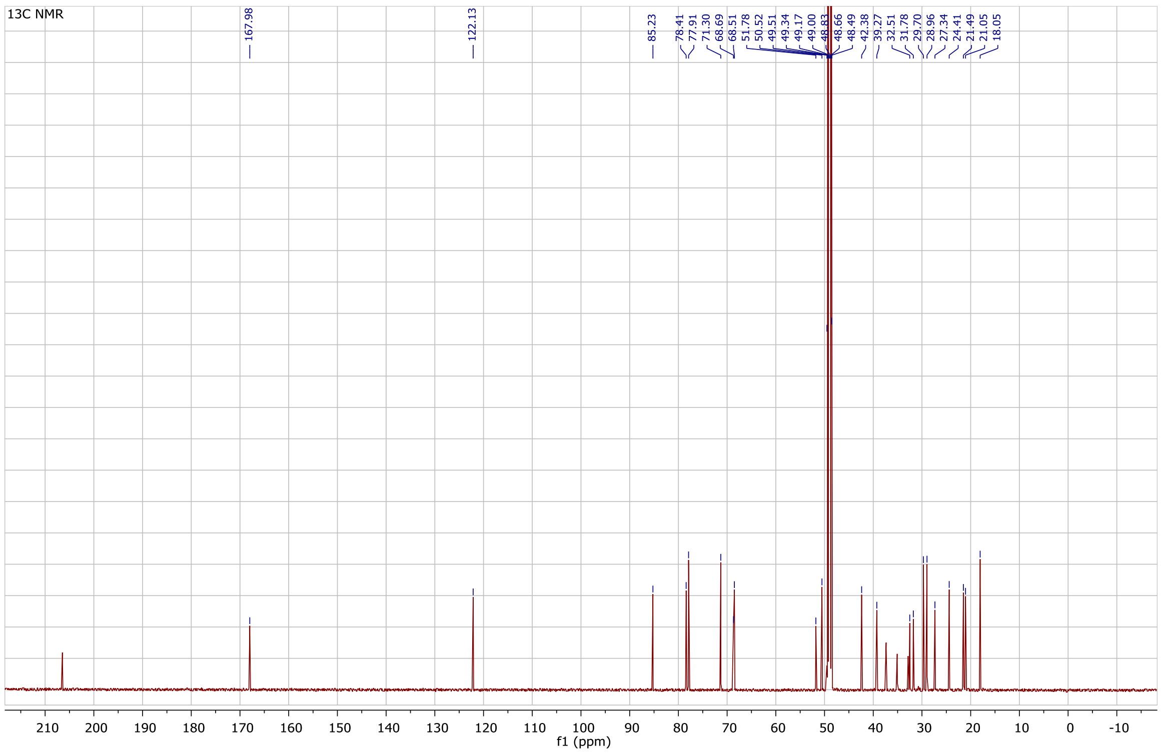

Figure S2. ^{13}C NMR spectrum (125 MHz, CD_3OD) of compound **1.**

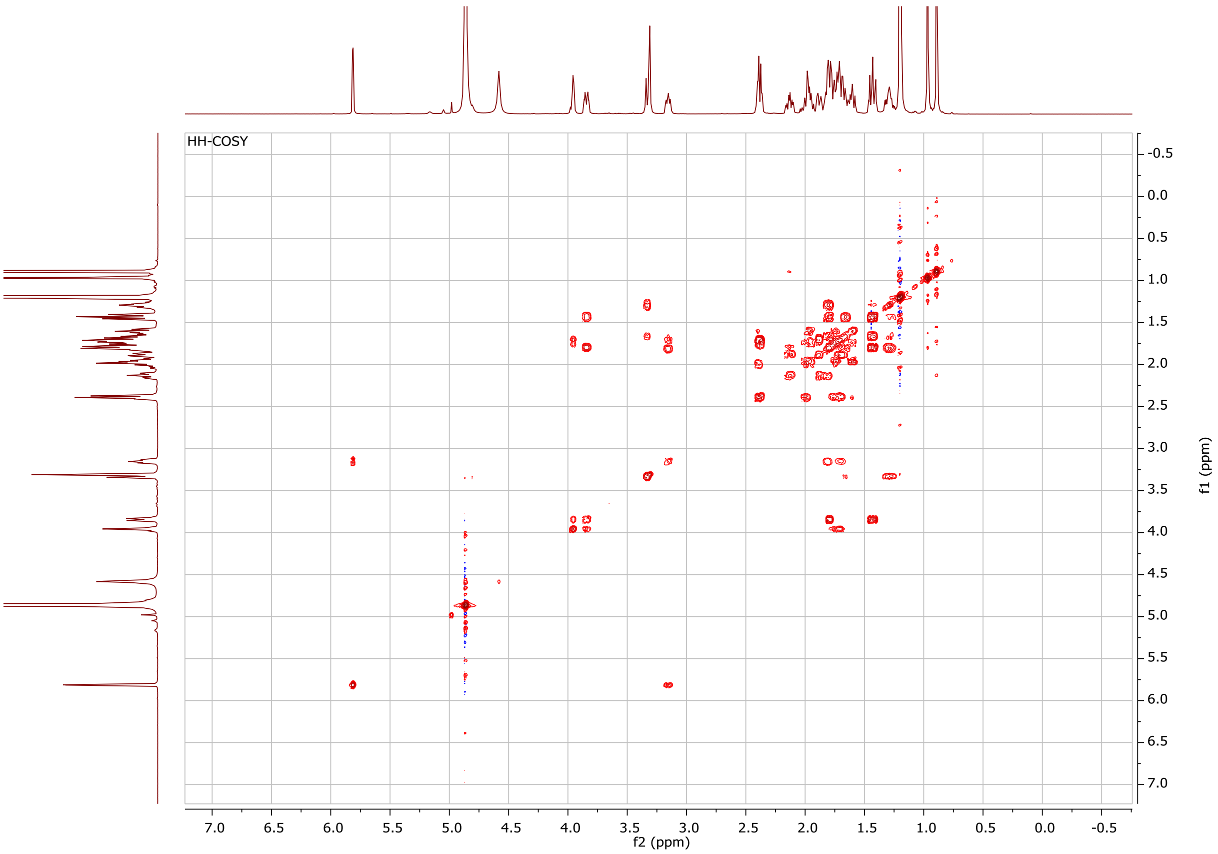

Figure S3. HH-COSY spectrum (500 MHz, CD3OD) of compound **1.**

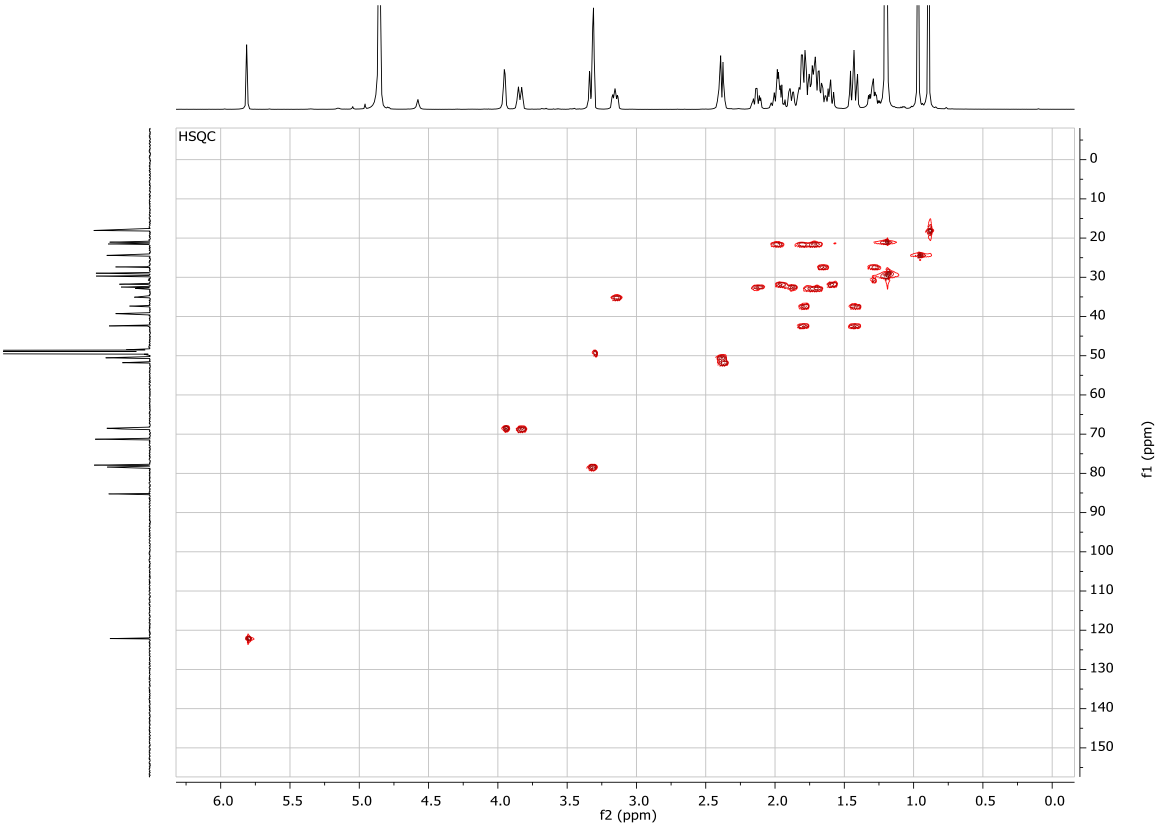

Figure S4. HSQC spectrum (500/125 MHz, CD3OD) of compound **1.**

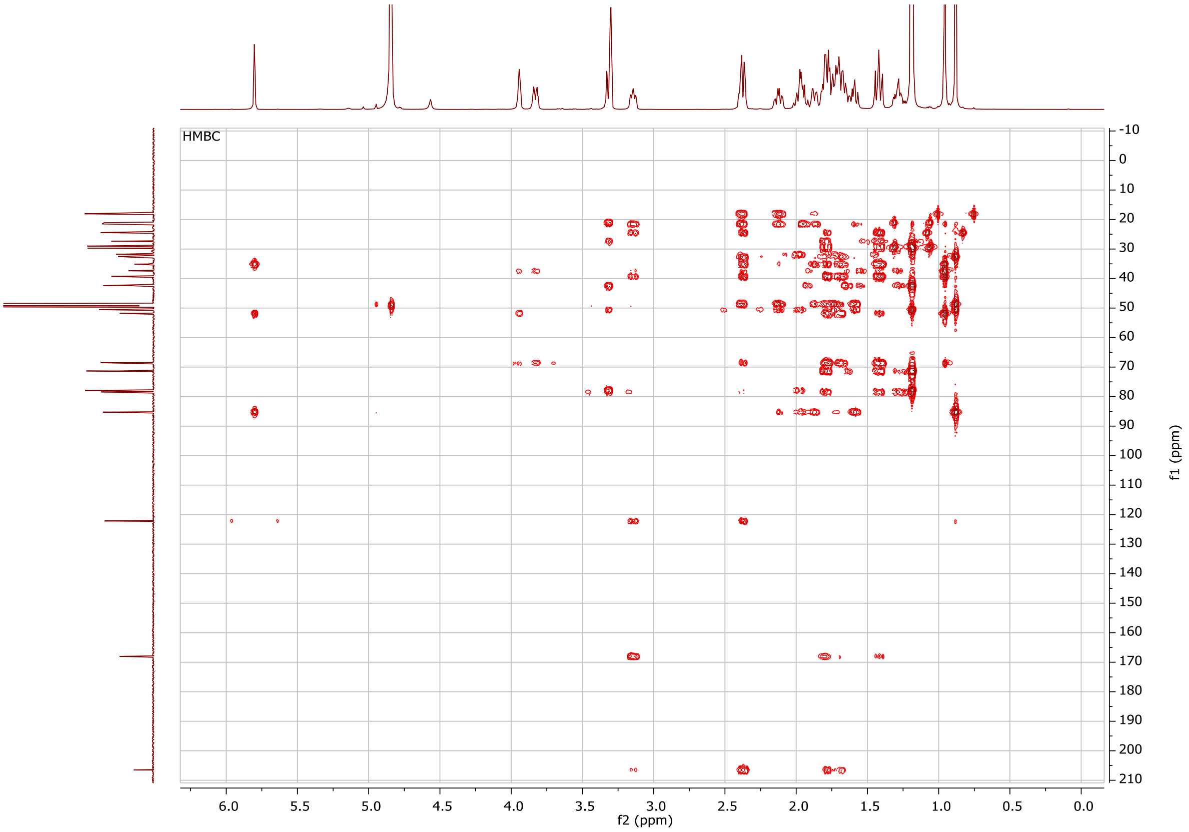

Figure S5. HMBC spectrum (500/125 MHz, CD3OD) of compound **1.**

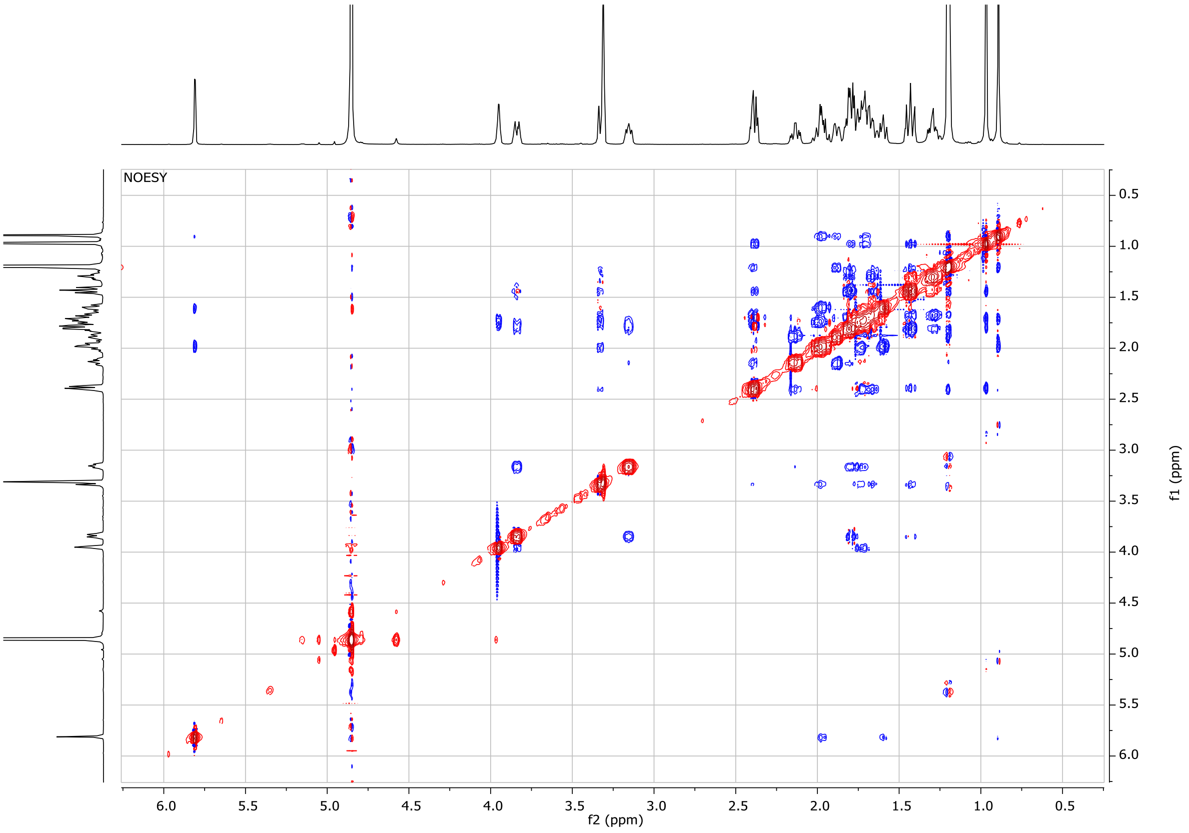

Figure S6. NOESY spectrum (500 MHz, CD3OD) of compound **1.**

Compound 2 (polypodine B)

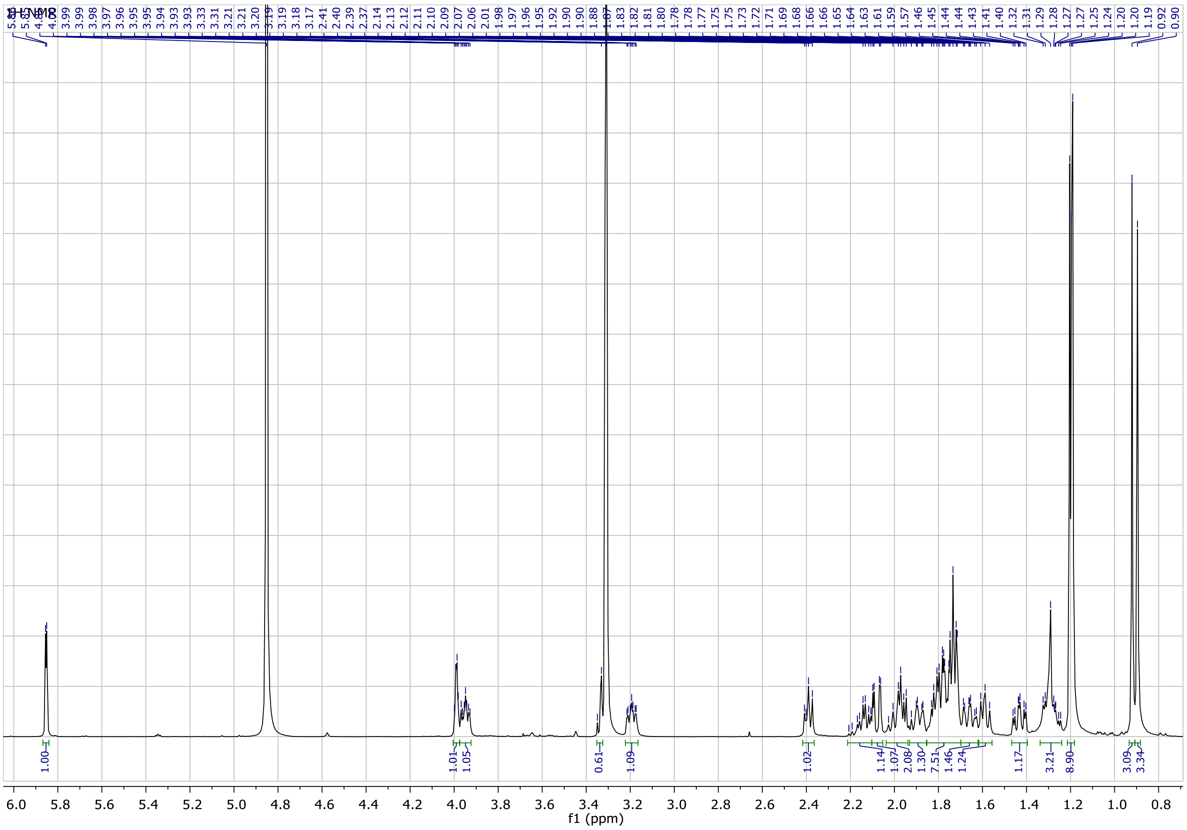

Figure S7. ^{1}H NMR spectrum (500 MHz, CD_3OD) of compound **2.**

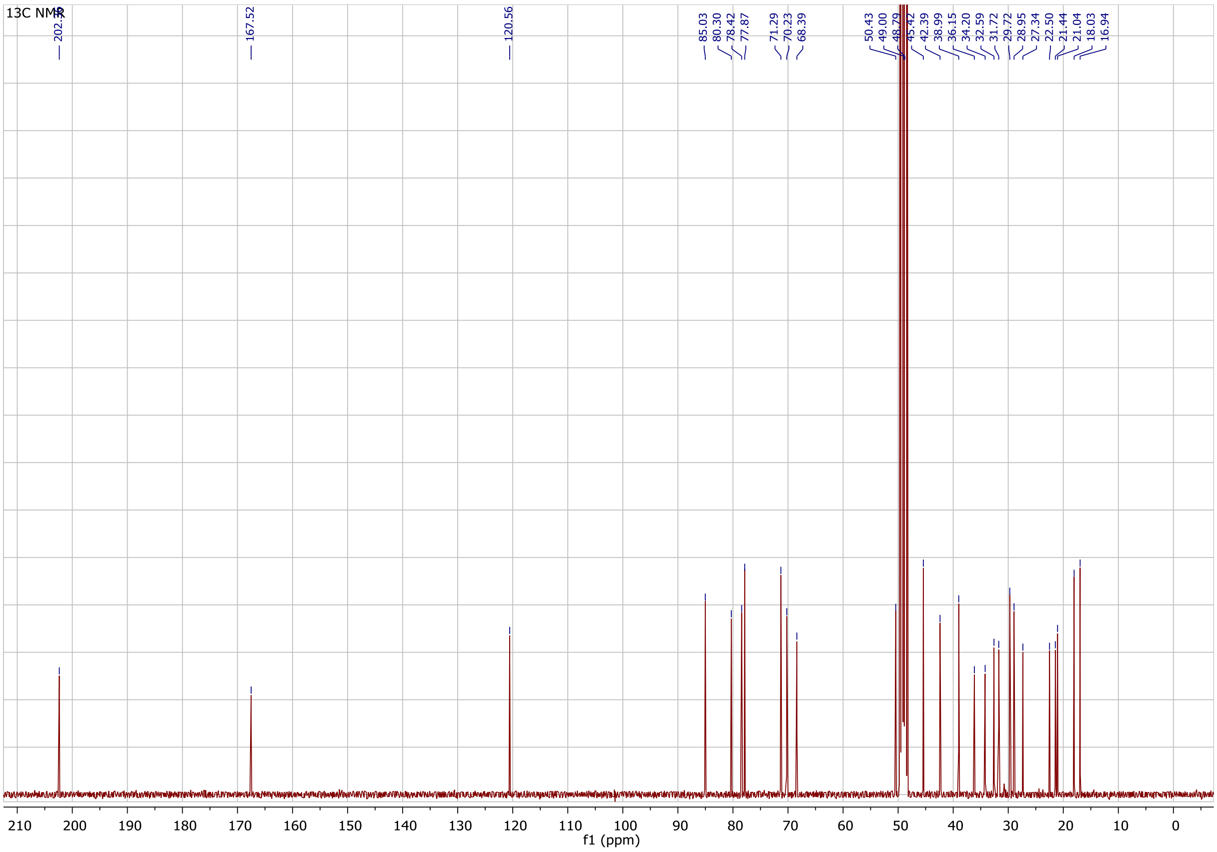

Figure S8. ^{13}C NMR spectrum (125 MHz, CD_3OD) of compound **2.**

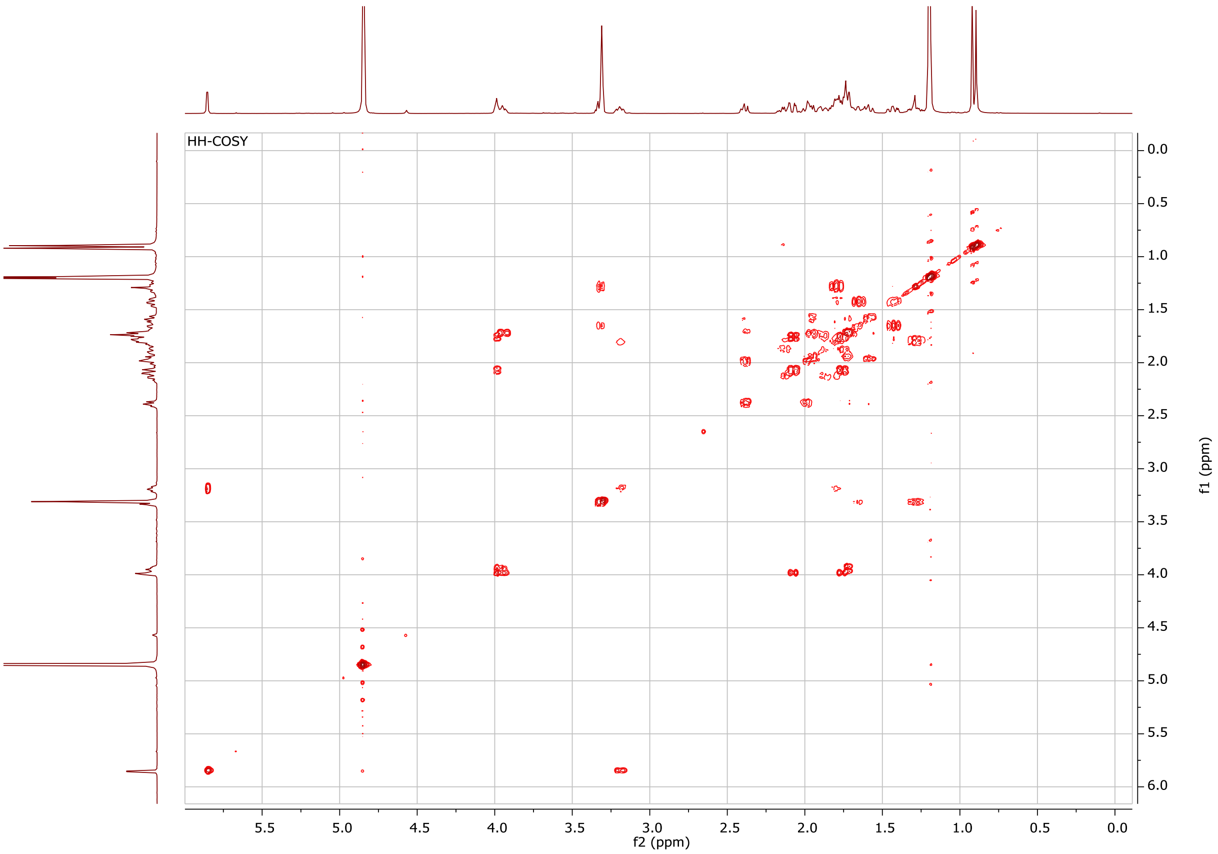

Figure S9. HH-COSY spectrum (500 MHz, CD3OD of compound **2.**

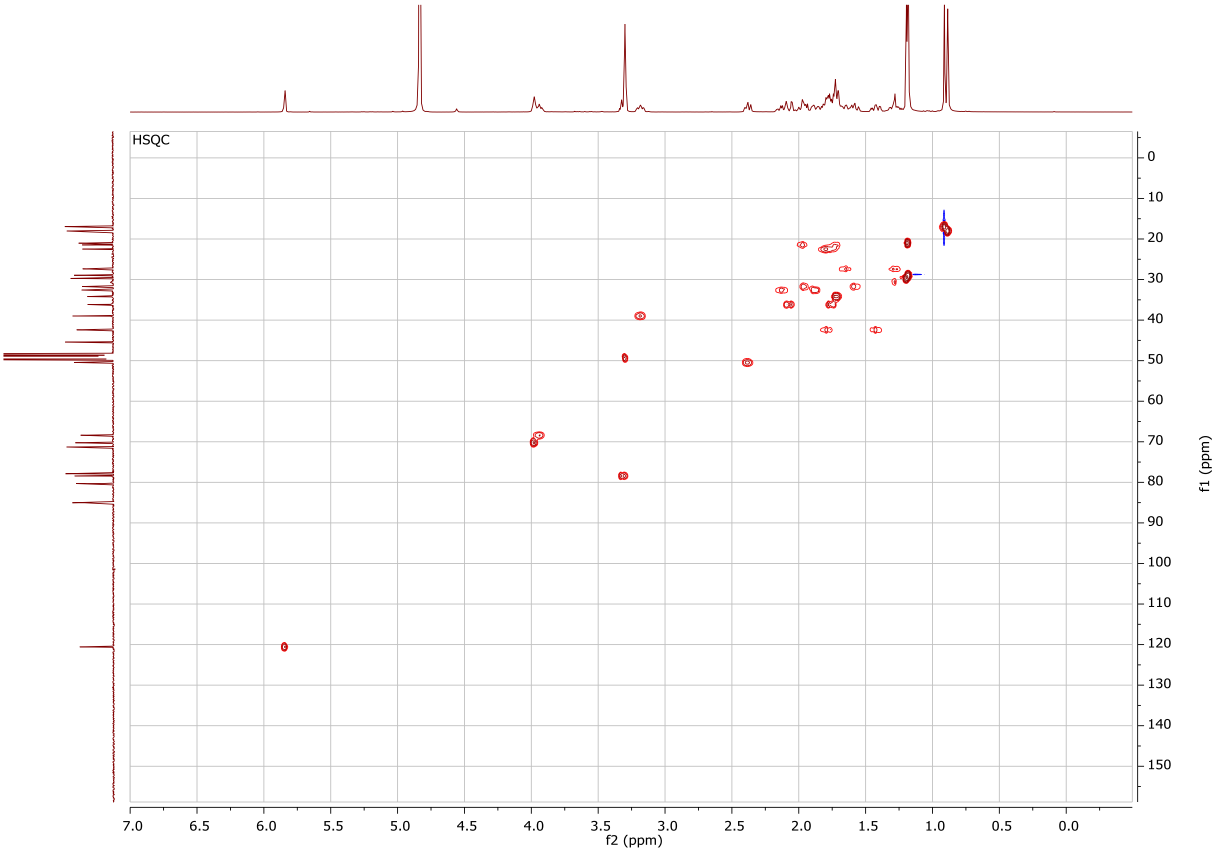

Figure S10. HSQC spectrum (500/125 MHz, CD3OD) of compound **2.**

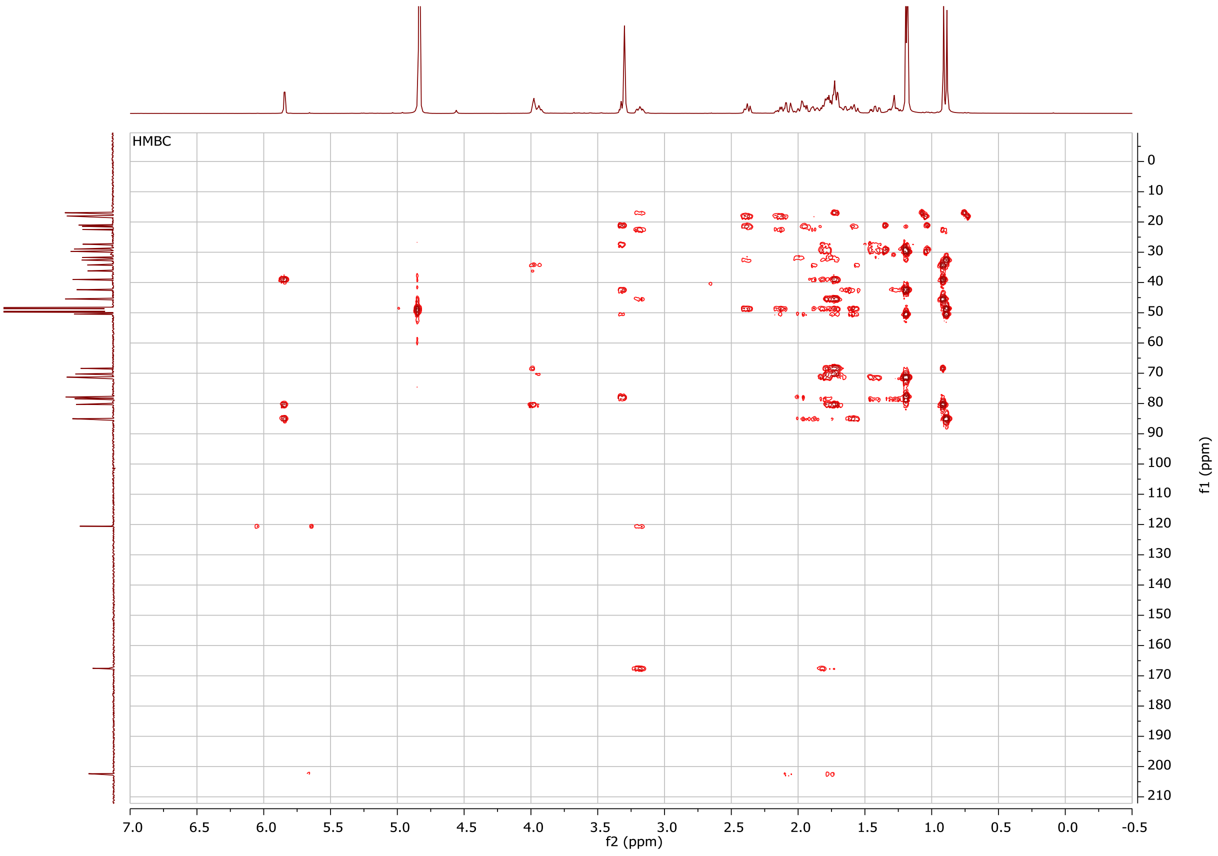

Figure S11. HMBC spectrum (500/125 MHz, CD_3OD) of compound **2.**

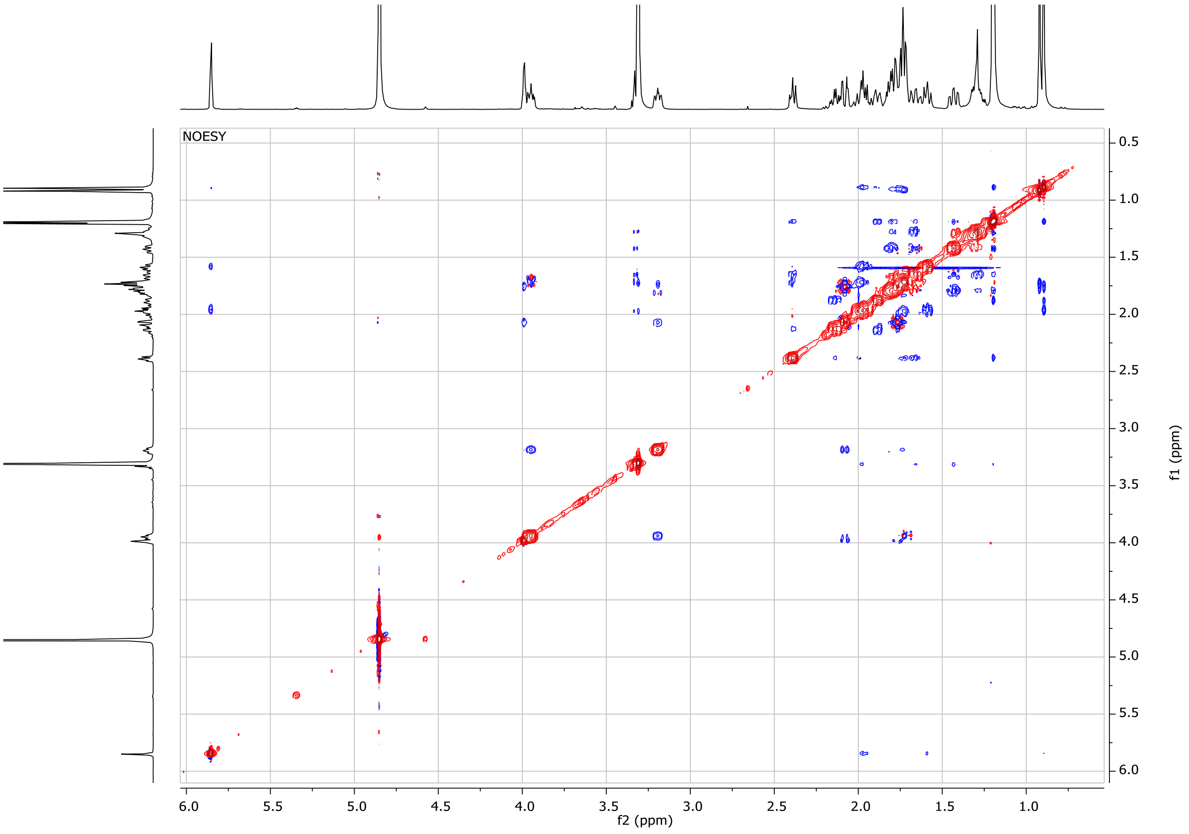

Figure S12. NOESY spectrum (500 MHz, CD_3OD) of compound **2.**

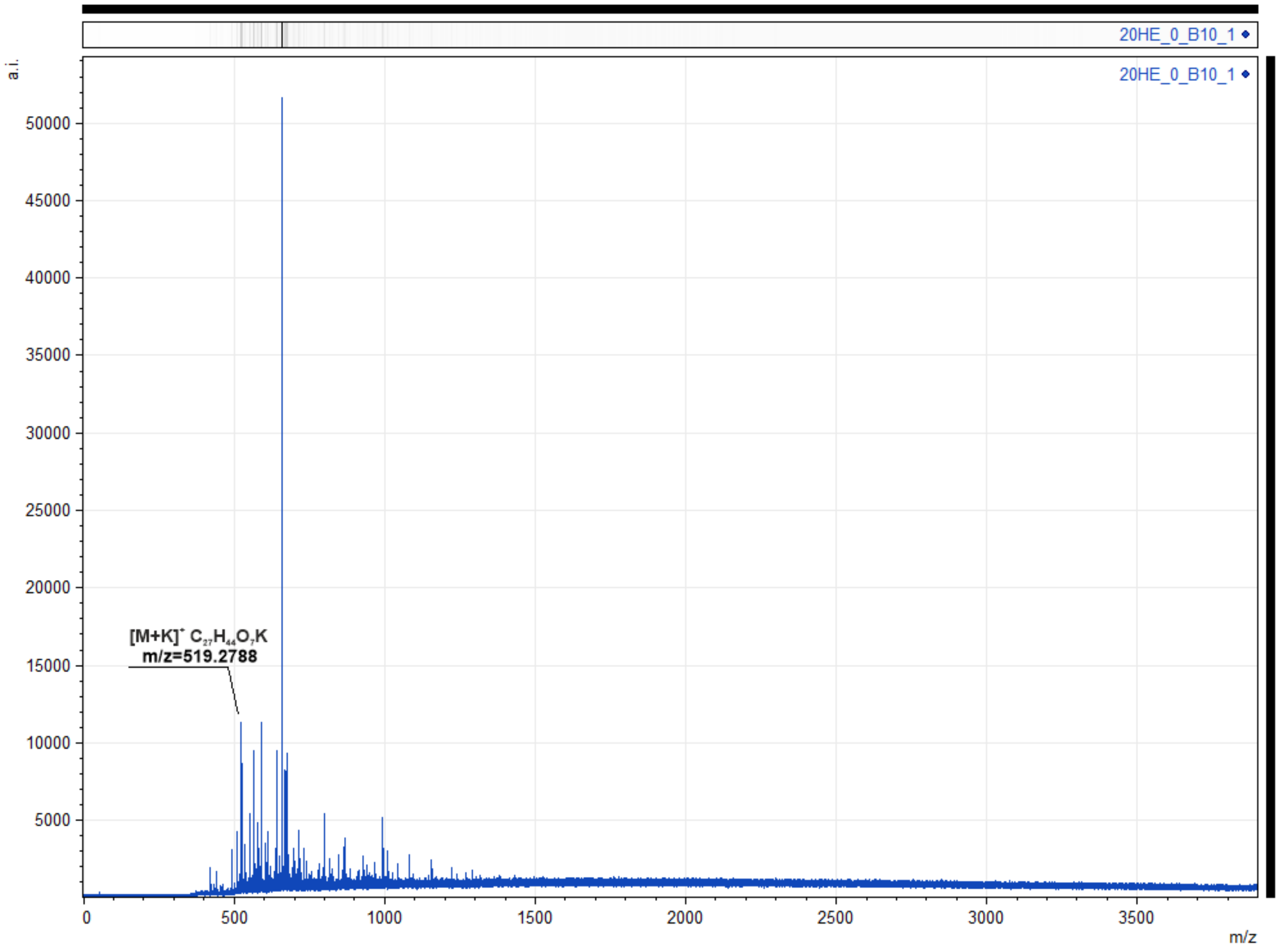

Figure S13. MALDI mass spectrum of 20-hydroxyecdysone (**1**) showing pseudo-molecular ion at m/z = 519.2788, $[M+K]^+$ $C_{27}H_{44}O_7K$ requires 519.2719.

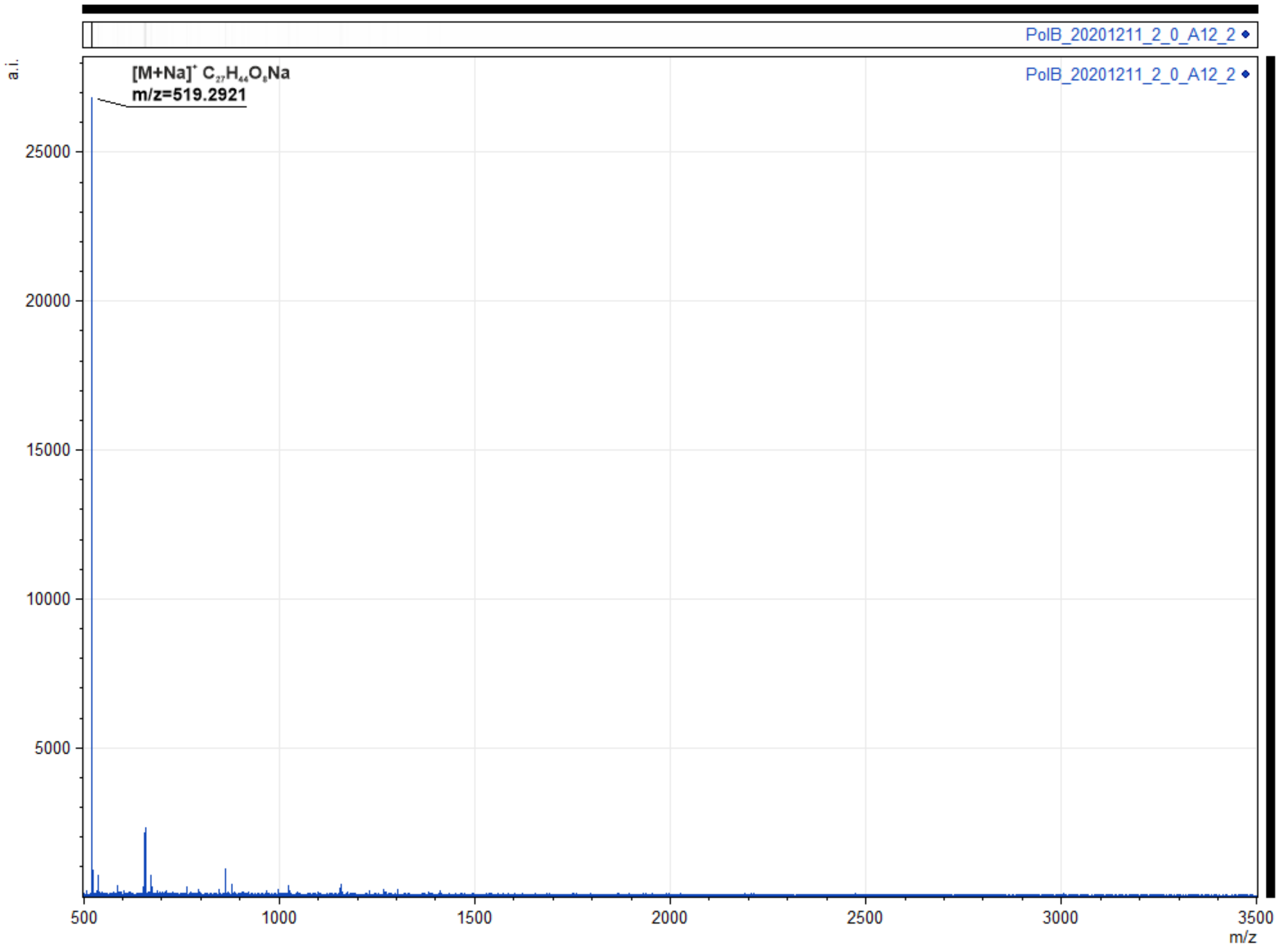

Figure S14. MALDI mass spectrum of polypodine B (**2**) showing pseudo-molecular ion at m/z = 519.2921, $[M+Na]^+$ $C_{27}H_{44}O_8Na$ requires 519.2928.

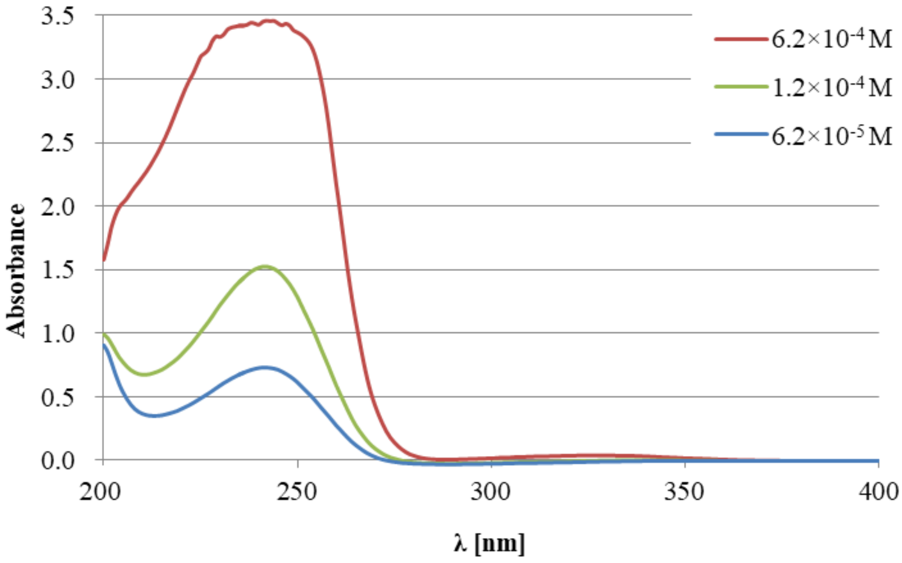

Figure S15. Concentration-dependent UV-Vis spectra of 20-hydroxyecdysone recorded in methanol. The concentration values are given in mol/dm^3; λ_{max} = 242 nm, logε = 4.07

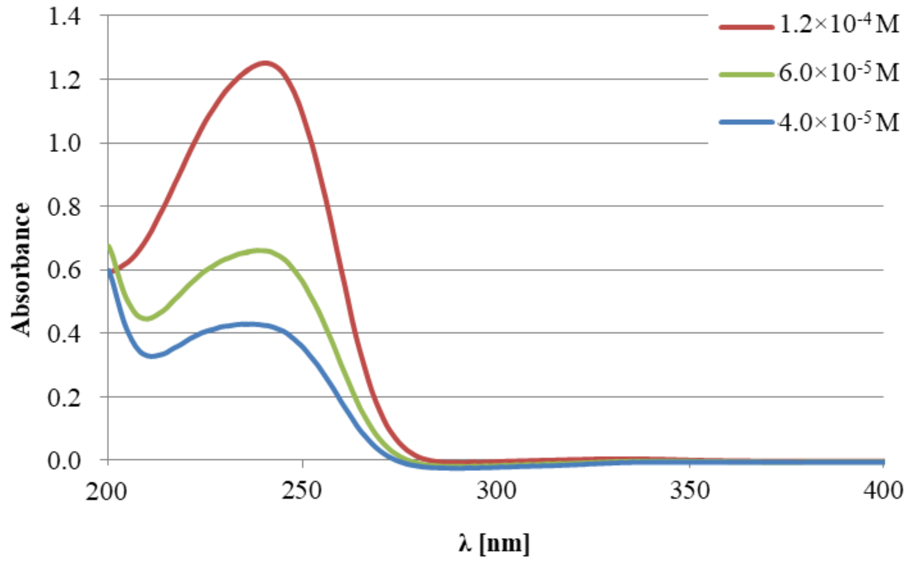

Figure S16. Concentration-dependent UV-Vis spectra of polypodine B recorded in ethanol. The concentration values are given in mol/dm^3; λ_{max} = 237 nm, logε = 4.03

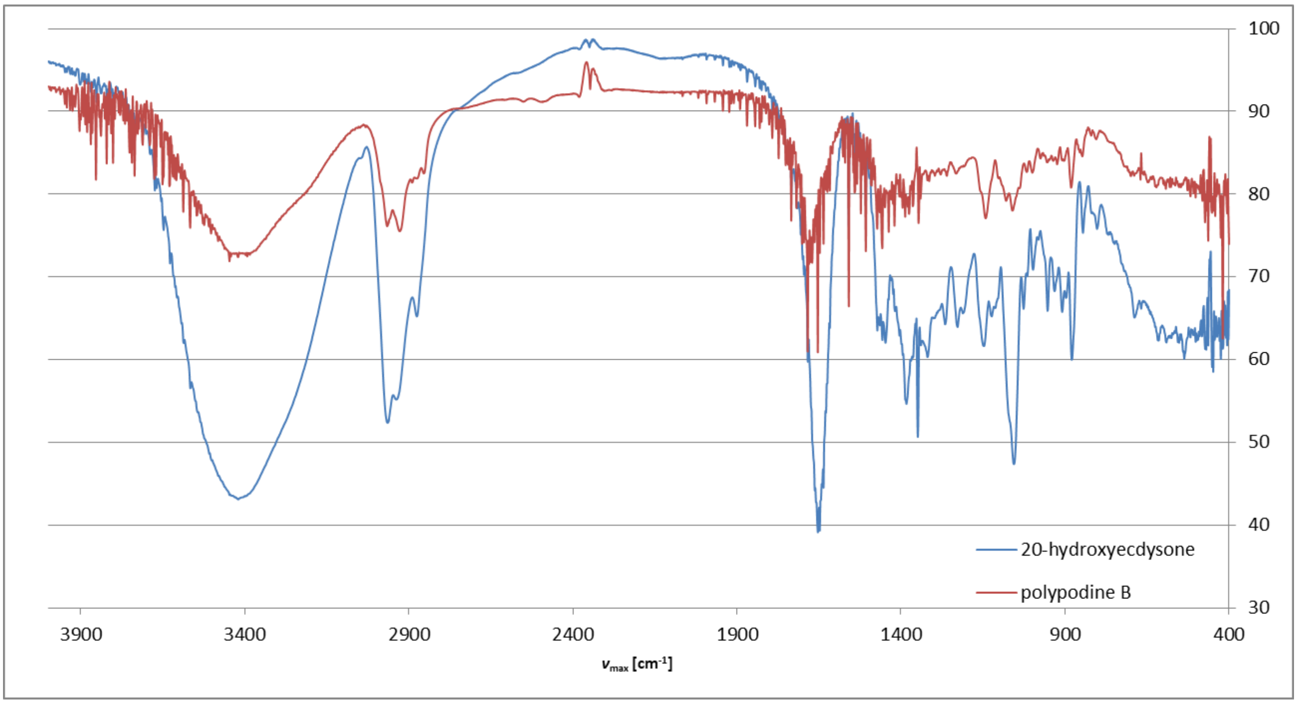

Figure S17. FT-IR spectra of 20-hydroxyecdysone (**1**); and polypodine B (**2**);

IR data for 20-hydroxyecdysone (KBr, vmax/cm–1): 2958s (CH3, CH2), 2927s (CH3, CH2), 2872s (CH3, CH2), 1678s (C=O) 1647s (C=C), 1635s (C=C), 1558s, 1379s (CH3, C-OH), 1348s (C-OH), 1313w (C-OH), 1261w (C-OH), 1224w (CH3, C-OH), 1141s (C-CH3), 1114w (C-C), 1051s (cyclohexane, CH2-OH), 1022w (cyclohexane) , 993w (C-C), 950w (C-H), 875s, 840w (C-C).

IR data for polypodine B (KBr, vmax/cm–1): 2933s (CH3, CH2), 2918s (CH3, CH2), 2848s (CH3, CH2), 1683s (C=O), 1652s (C=C), 1635s (C=C), 1558s, 1346s (C-OH), 1338w (C-OH), 1307w (C-OH), 1302w (C-OH), 1286w (C-OH), 1271w (C-OH), 1253w (C-OH), 1223w (CH3, C-OH), 1136s (C-CH3), 1070s (cyclohexane, CH2-OH), 1053s (cyclohexane, CH2-OH), 1012w (cyclohexane), 993w (C-C), 877s, 842w (C-C).

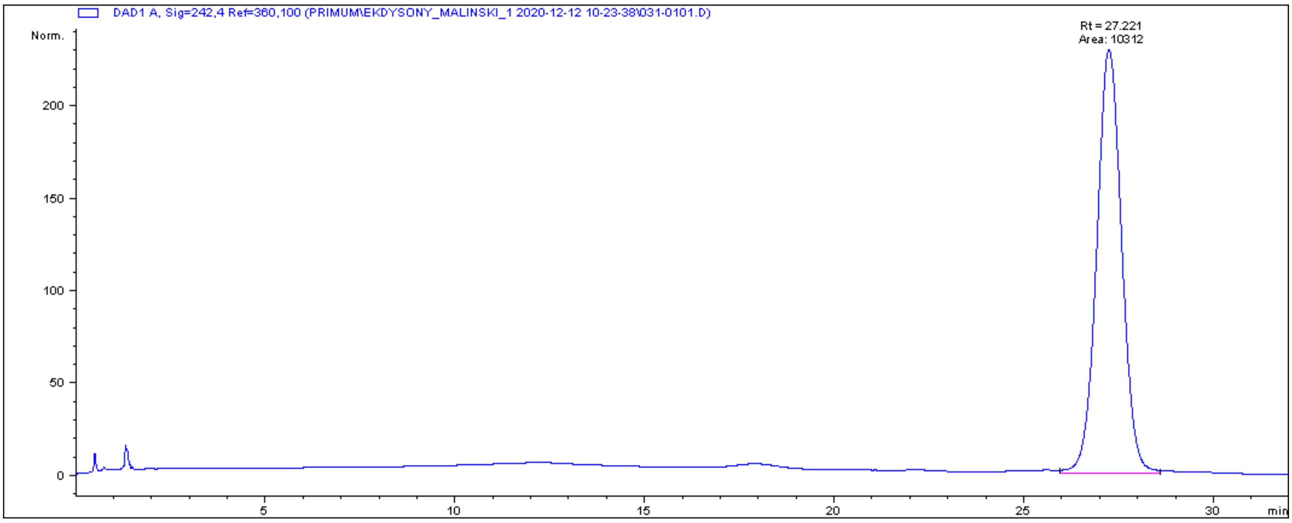

Figure S18. HPLC-DAD chromatogram of isolated 20-hydroxyecdysone (**1**).

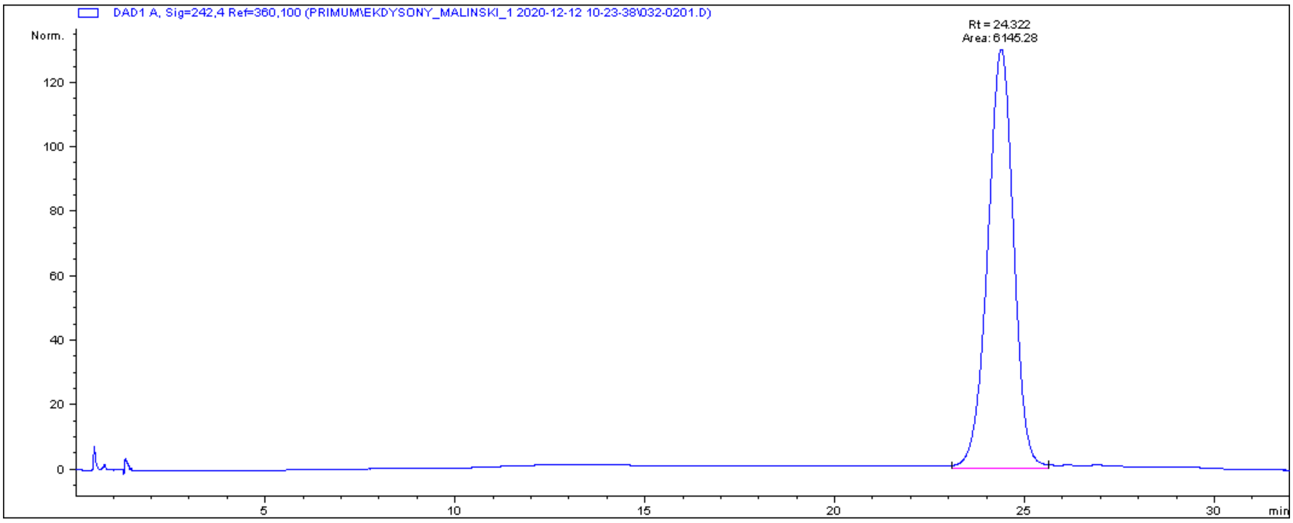

Figure S19. HPLC-DAD chromatogram of isolated polypodine B (**2**).

Quantitative evaluation of ecdysteroids in fractions

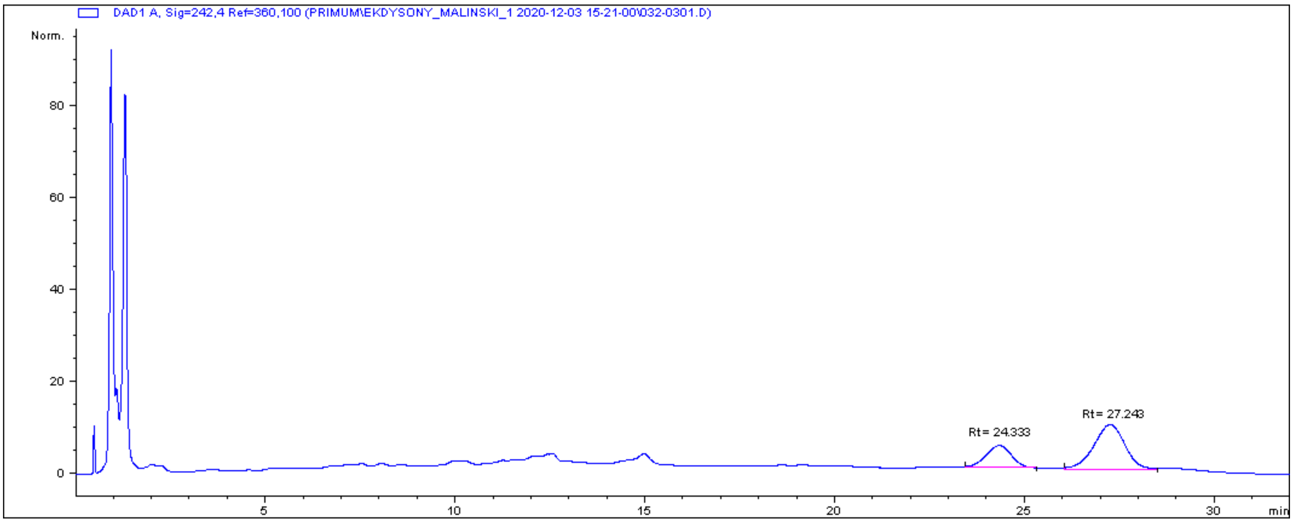

Figure S20. HPLC-DAD chromatogram of 40% methanolic root fraction showing 20-hydroxyecdysone (**1**) and polypodine B (**2**).

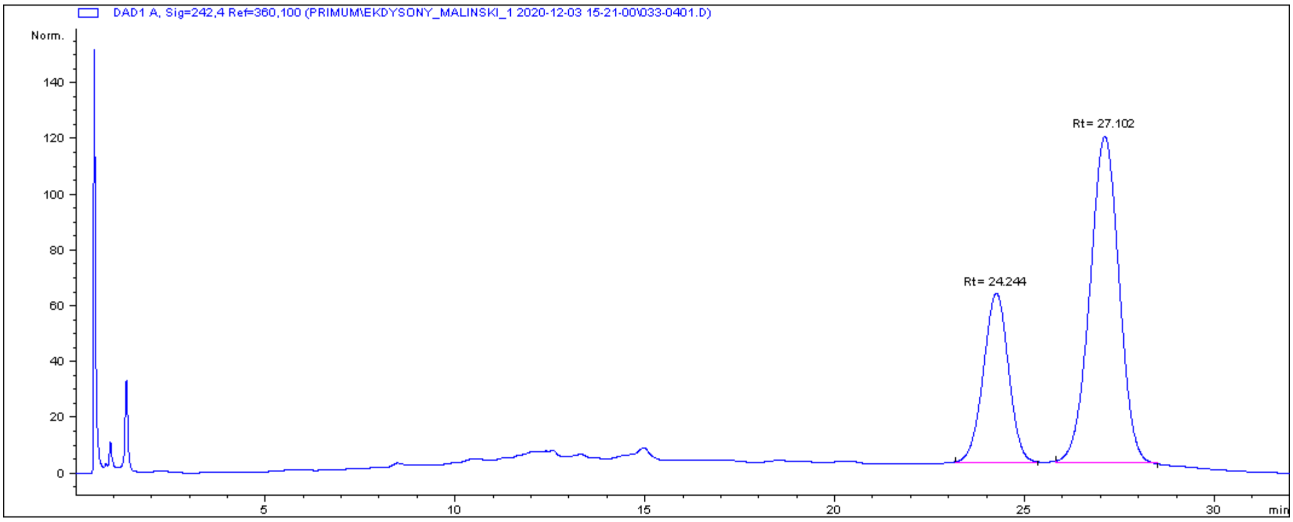

Figure S21. HPLC-DAD chromatogram of 80% methanolic root fraction showing 20-hydroxyecdysone (**1**) and polypodine B (**2**).

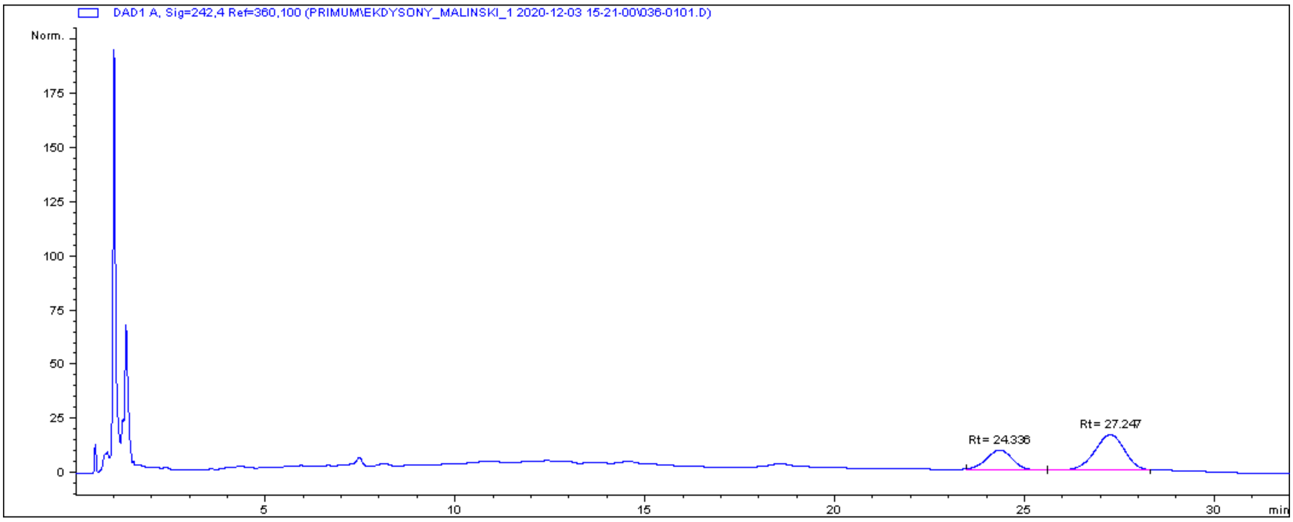

Figure S22. HPLC-DAD chromatogram of 40% methanolic herb fraction showing 20-hydroxyecdysone (**1**) and polypodine B (**2**).

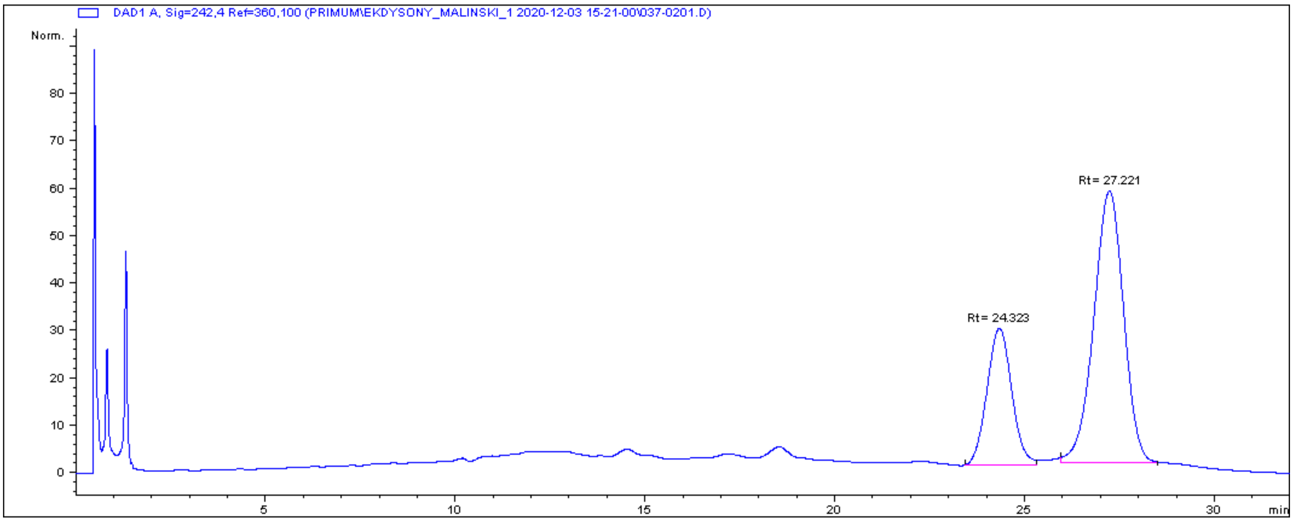

Figure S23. HPLC-DAD chromatogram of 80% methanolic herb fraction showing 20-hydroxyecdysone (**1**) and polypodine B (**2**).

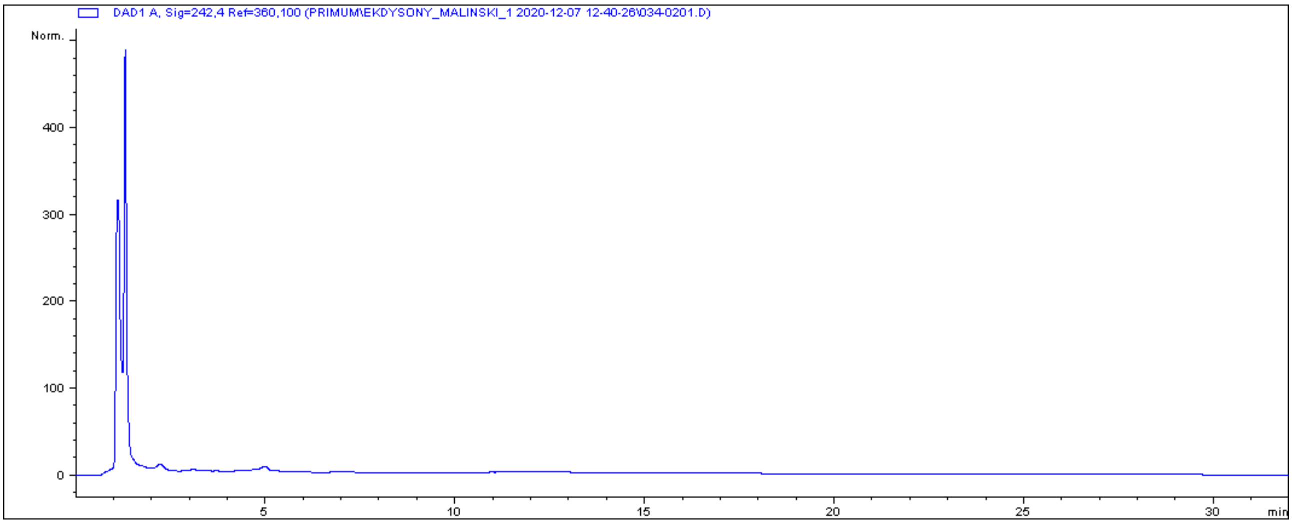

Figure S24. HPLC-DAD chromatogram of 40% methanolic callus fraction showing no ecdysteroids.

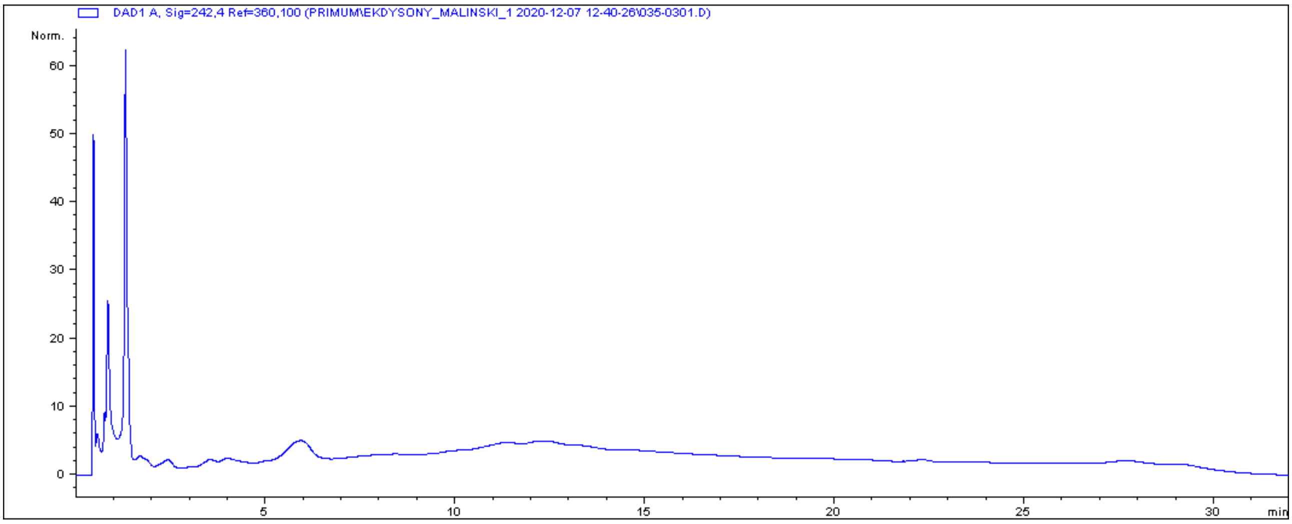

Figure S25. HPLC-DAD chromatogram of 80% methanolic callus fraction showing no ecdysteroids.

Additional Experimental Data

Table S1. The effect of nystatin (positive control) on *Acanthamoeba* trophozoite inhibition during three-day treatment.

Nystatin concentration [mg/mL]	Duration of treatment [days]		
	1st day	2nd day	3rd day
	MT ± SD	MT ± SD	MT ± SD
control	5.89 ± 2.05	14.25 ± 3.03	19.94 ± 3.55
0.05	2.93 ± 1.01*	2.28 ± 0.80*	8.44 ± 1.42*
0.1	2.76 ± 1.00*	1.39 ± 0.83*	3.66 ± 2.09*
0.2	2.61 ± 0.95*	1.17 ± 0.89*	2.56 ± 0.76*

MT - mean trophozoite number in haemocytometer chamber * P<0.05 statistically significant difference in comparison with the control during the same time interval; n=18

Table S2. The toxicity of studied 40% and 80% aqueous methanolic fractions from *Lychnis flos-cuculi* extracts and isolated ecdysteroids against *Aliivibrio fischeri* after 5 and 15 minutes, measured with the use of Microtox assay.

Sample	Sample dilution [mg/mL]	Ecdysteroid content [μg/mL]		Cell viability decrease [%]	
		20E	polB	t = 5 min	t = 15 min
Root, 40% fraction	5	15	12.3	56.51%	63.27%
	0.5	1.5	1.2	15.76%	20.12%
Root, 80% fraction	5	184.4	124.4	99.30%	99.99%
	2.5	92.2	62.2	88.01%	95.65%
	0.5	18.4	12.4	49.50%	52.39%
	0.05	1.8	1.2	29.72%	31.48%
Herb, 40% fraction	5	25.5	22.7	88.43%	88.75%
	0.5	2.5	2.3	29.14%	35.59%
Herb, 80% fraction	5	91.3	57.2	99.93%	99.93%
	2.5	45.7	28.6	99.96%	99.94%
	0.5	9.1	5.7	68.31%	68.03%
	0.05	0.9	0.6	35.08%	36.34%
Callus, 40% fraction	5	0	0	99.50%	99.79%
	0.5	0	0	42.89%	45.86%
Callus, 80% fraction	5	0	0	99.93%	99.93%
	2.5	0	0	99.94%	99.94%
	0.5	0	0	60.17%	59.24%
	0.05	0	0	36.57%	37.81%
20-hydroxyecdysone (**1**)	1	1000	0	57.54%	60.22%
	0.5	500	0	46.58%	51.43%
Polypodine B (**2**)	2	0	2000	69.18%	72.42%
	1	0	1000	26.60%	29.81%
	0.5	0	500	6.50%	12.98%

Abbreviations: 20E – 20-hydroxyecdysone, polB – polypodine B

Table S3. Concentration of 20-hydroxyecdysone and polypodine B in respective dilutions of different *Lychnis flos-cuculi* material samples used in antiamoebic activity bioassay.

Sample	Sample dilution [mg/mL]	Ecdysteroid concentration* [μg/mL]	Peak amoeba growth inhibition [%]
Herb extract	1	25.07	19.20
	5	125.35	26.23
	10	250.70	47.08
Root extract	1	37.48	30.92

	5	187.40	47.09
	10	374.80	91.10
Root 40% fraction	0.5	2.72	26.52
	2.5	13.60	42.94
	5	27.20	87.27
Root 80% fraction	0.05	3.09	19.62
	0.1	6.18	73.79
	0.5	30.88	88.64
Herb 40% fraction	0.1	0.96	11.01
	0.5	4.81	12.79
	1	9.62	18.00
Herb 80% fraction	0.1	2.97	28.31
	0.5	14.85	38.07
	1	29.70	41.10
Callus, 40% fraction	0.5	0	39.96
	1	0	73.88
	5	0	91.27
Callus, 80% fraction	0.2	0	25.52
	1	0	42.94
	2	0	90.40
20-hydroxyecdysone**	0.05	50	41.72
	0.1	100	62.55
	0.5	500	97.87
Polypodine B**	0.05	50	36.12
	0.1	100	56.88
	0.5	500	97.27

* sum of 20-hydroxyecdysone and polypodine B, ** single compounds
